# A Comparison of Penalized Maximum Likelihood Estimation and Markov Chain Monte Carlo Techniques for Estimating Confirmatory Factor Analysis Models With Small Sample Sizes

**DOI:** 10.3389/fpsyg.2021.615162

**Published:** 2021-04-29

**Authors:** Oliver Lüdtke, Esther Ulitzsch, Alexander Robitzsch

**Affiliations:** ^1^IPN – Leibniz Institute for Science and Mathematics Education, Kiel, Germany; ^2^Centre for International Student Assessment, Kiel, Germany

**Keywords:** measurement error, latent variable models, Bayesian methods, prior distribution, Markov Chain Monte Carlo, penalized maximum likelihood estimation, constrained maximum likelihood estimation, confirmatory factor analysis

## Abstract

With small to modest sample sizes and complex models, maximum likelihood (ML) estimation of confirmatory factor analysis (CFA) models can show serious estimation problems such as non-convergence or parameter estimates outside the admissible parameter space. In this article, we distinguish different Bayesian estimators that can be used to stabilize the parameter estimates of a CFA: the mode of the joint posterior distribution that is obtained from penalized maximum likelihood (PML) estimation, and the mean (EAP), median (Med), or mode (MAP) of the marginal posterior distribution that are calculated by using Markov Chain Monte Carlo (MCMC) methods. In two simulation studies, we evaluated the performance of the Bayesian estimators from a frequentist point of view. The results show that the EAP produced more accurate estimates of the latent correlation in many conditions and outperformed the other Bayesian estimators in terms of root mean squared error (RMSE). We also argue that it is often advantageous to choose a parameterization in which the main parameters of interest are bounded, and we suggest the four-parameter beta distribution as a prior distribution for loadings and correlations. Using simulated data, we show that selecting weakly informative four-parameter beta priors can further stabilize parameter estimates, even in cases when the priors were mildly misspecified. Finally, we derive recommendations and propose directions for further research.

## Introduction

In the social and behavioral sciences, constructs (e.g., intelligence, extraversion) are often conceptualized as latent variables that are measured by error-prone observed indicators (e.g., items). Structural equation modeling (SEM) is a very prominent approach that is used to correct for measurement error when assessing multivariate relationships among latent constructs (Bollen, 1989; Hoyle, 2012). In the SEM approach, a measurement part is distinguished from a structural part. In the measurement part, measurement models are specified to allow for an error-free estimation of the relations in the structural model. In research practice, maximum likelihood (ML) estimation is routinely used to obtain parameter estimates for structural equation models. However, one major limitation of ML estimation is that it needs large sample sizes to reveal its optimal properties (e.g., unbiasedness, efficiency). With small to modest sample sizes and complex models, ML estimation can show serious estimation problems such as non-convergence or parameter estimates that are outside the admissible parameter space (e.g., negative variances; see Anderson and Gerbing, 1984; Boomsma, 1985; Hoogland and Boomsma, 1998; Chen et al., 2001; Gagné and Hancock, 2006; Wolf et al., 2013; Smid and Rosseel, 2020).

In the last decades, several researchers have shown that the Bayesian approach has the potential to solve some of the estimation problems that occur in small sample applications of SEM (e.g., Lee, 2007; Song and Lee, 2009; Kaplan and Depaoli, 2012; Muthén and Asparouhov, 2012). First, if appropriate prior distributions are used, the Bayesian approach guarantees that parameter estimates will be within the admissible range, and estimation problems can usually be avoided. Second, Bayesian methods allow for the stabilization of parameter estimates by specifying weakly informative prior distributions for the SEM parameters (Lee and Song, 2004; Chen et al., 2014; Can et al., 2015; Depaoli and Clifton, 2015; McNeish, 2016; Lüdtke et al., 2018; van Erp et al., 2018; Miocevic et al., 2020). The basic idea is that incorporating even a small amount of information into the prior distribution of the SEM parameters provides some direction for their estimation, while inferences can still be driven by the data (Gelman et al., 2014).

In this article, we focus on the estimation of confirmatory factor analysis (CFA) models in which several latent factors are measured by a set of observed variables (Bollen, 1989). We investigate two critical issues in the Bayesian estimation of CFA models with small sample sizes. First, we discuss different Bayesian point estimators that can be used as estimates for CFA model parameters: the mode of the joint posterior, and the mode, mean, or median of the marginal posterior. Furthermore, we clarify that two popular methods for calculating Bayesian point estimates, penalized maximum likelihood (PML) estimation and Markov Chain Monte Carlo (MCMC) methods, produce different Bayesian point estimates and compare the performance of the different Bayesian point estimators to the traditional ML estimation of CFA models. Second, we discuss the specification of prior distributions in the Bayesian approach and argue that it can be advantageous to choose a parameterization in which the model parameters (i.e., standardized loadings, latent correlations) are bounded. More specifically, we suggest the four-parameter beta distribution as a prior distribution for bounded parameters (see also Muthén and Asparouhov, 2012; Merkle and Rosseel, 2018) and investigate in a simulation study how the specification of weakly informative prior distributions can help to stabilize parameter estimates in small sample size conditions.

The article is organized as follows. We start by describing how a basic CFA model is estimated with traditional ML estimation. We then discuss the specification of CFA models in the Bayesian approach and describe different Bayesian estimators that can be used to estimate CFA model parameters. In the context of a CFA model with two latent factors, we discuss issues of parameterization and the specification of prior distributions, and we illustrate conditions under which the different Bayesian estimators produce different results. We then present the results of two simulation studies in which we compare traditional ML estimation with the Bayesian approach. In the first simulation study, we evaluate the influence of correctly and misspecified prior distributions on the quality of parameter estimates in small sample size conditions. In the second simulation study, we investigate the robustness of the Bayesian approach against distributional misspecifications (i.e., non-normality). Finally, we derive recommendations and propose directions for further research.

## Confirmatory Factor Analysis

Let **x** denote a vector of *p* observed variables. Then, a CFA model with *m* latent factors is represented as follows:

(1) x=ν+Λ⁢η+ε,

where **ν** is a *p* × 1 vector containing intercepts, **Λ** is a *p* × *m* matrix of factor loadings, **η** is an *m* × 1 vector of latent factors, and **ε** denotes the vector of multivariate normally distributed residuals with zero mean vector and covariance matrix **Ω**. In the following, we assume that the mean structure is saturated and completely reflected in the intercepts, that is, E(**x**) = **ν**. Thus, the focus is on modeling the covariance structure **Σ** of the observed variables.

The covariance matrix of the observed variables **Σ** can be written as a function of the model parameters of the CFA model:

(2) Σ(θ)=ΛΦΛ+TΩ,

Where **Φ** is the *m* × *m* covariance matrix of the latent factors, θ = (θ_1_,…,θ_*q*_) is a *q* × 1 vector that contains the *q* non-redundant parameters in **Λ**, **Φ**, and **Ω** that are estimated; and **Σ**(**θ**) is the model-implied covariance matrix. Thus, the covariance of the observed variables can be decomposed into a part due to the covariance structure of the latent factors and a part that is due to measurement error.

### Maximum Likelihood Estimation

Maximum Likelihood estimation is routinely used to obtain parameter estimates of CFA models (Jackson et al., 2009). Let **x**_1_,…,**x***_*n*_* denote a set of independently and identically distributed *p* × 1 vectors of observed variables that are multivariate normally distributed. Then, for an observed data set, **X** = {**x***_*i*_*}_*i=1,…,n*_, the likelihood function is written as:

(3) $$L\,\left(\theta |\text{X}\right)=\prod_{i=1}^{n}f\,\left({\text{x}}_{i};\nu ,\Sigma \,\left(\theta \right)\right),$$

where *f*(**x**; **μ**, **Σ**) denotes the multivariate normal density with mean vector μ and covariance matrix **Σ**. It is known that the sample-based covariance matrix $\text{S}=\frac{1}{n}\,\sum_{i=1}^{n}\left({\text{x}}_{i}-\bar{\text{x}}\right)\,{\left({\text{x}}_{i}-\bar{\text{x}}\right)}^{\prime }$ is a sufficient statistic for **Σ**, and hence for **Σ**(θ), which also implies sufficiency for θ. Thus, the likelihood can be written as *L*(θ| **X**) = *L*(θ|**S**), and the sample covariance matrix **S** of the *p* observed variables can be used as input in the SEM framework. The log-likelihood can be simplified as (Bollen, 1989):

(4) $$l\,\left(\theta \right)=\log L\,\left(\theta |\text{S}\right)=-\frac{n}{2}\,\left[p\cdot \log\left(2\,\pi \right)+\text{log}\,\left|\Sigma \,\left(\theta \right)\right|+\text{tr}\,\left(\Sigma \,{\left(\theta \right)}^{-1}\,\text{S}\right)\right],$$

where tr is the trace operator, that is, the sum of the diagonal elements of a square matrix. The value ${\hat{\theta }}_{\text{ML}}=\underset{\theta }{\arg\,\max}l\,\left(\theta \right)$ that maximizes *l*(θ) is the ML estimate. It should be emphasized that the latent variables η do not appear in the likelihood in Equation 4. Therefore, it has also been referred to as the marginal likelihood where the latent variables are integrated out (Fox et al., 2017; Merkle et al., 2019).

Statistical inference in ML estimation is based on the asymptotic covariance matrix of the ML estimator ${\hat{\theta }}_{\text{ML}}$ which is obtained from the negative second partial derivatives of the log-likelihood function with respect to the model parameters:

(5) $$\mathrm{ACOV}\,\left({\hat{\theta }}_{\text{ML}}\right)={\left\{-{\left[\frac{\partial^{2}l\,\left(\theta \right)}{\partial \theta \,\partial \theta^{\prime }}\right]|}_{\theta ={\hat{\theta }}_{\text{ML}}}\right\}}^{-1},$$

where the diagonal elements of the *q*×*q* matrix are used as estimates of standard errors. The term in brackets is also known as observed information matrix (with ${\hat{\theta }}_{\text{ML}}$ plugged into the matrix of the second partial derivatives of *l*(θ); see Held and Bové, 2014). In research practice, robust standard error estimates are often used for statistical inference in SEM (Savalei, 2014; Maydeu-Olivares, 2017).

The desirable properties of ML estimation (e.g., most efficient estimates) are based on asymptotic theory and are only guaranteed to hold with large sample sizes (Yuan and Bentler, 2007). In small samples and complex models, ML estimation is prone to serious estimation problems such as failure to converge or inadmissible solutions (e.g., negative variance estimates or correlations that are larger than one; Anderson and Gerbing, 1984; Wothke, 1993; Chen et al., 2001; Yuan and Chan, 2008). Furthermore, in small to medium samples, SEMs that correct for measurement error, even though approximately unbiased, can produce much more variable estimates of structural relationships (i.e., larger empirical sampling variance) than biased manifest approaches that ignore measurement error and use manifest scale scores (Hoyle and Kenny, 1999; Ledgerwood and Shrout, 2011; Savalei, 2019; see also Li and Beretvas, 2013; Zitzmann et al., 2016).

### Constrained Maximum Likelihood Estimation

As mentioned above, in standard ML estimation, parameter estimates are not constrained to any specific interval, and nothing prevents, for example, variance estimates from becoming negative (Savalei and Kolenikov, 2008; Held and Bové, 2014). *Constrained* ML estimation can mitigate estimation problems and avoid parameter estimates outside the admissible parameter space. For example, Lüdtke et al. (2018) showed in simulation studies that constrained ML estimation of the trait-state-error model for multi-wave data (Kenny and Zautra, 1995) outperformed unconstrained ML estimation in terms of the frequency of estimation problems and the accuracy of the parameter estimates (see also Gerbing and Anderson, 1987; Chen et al., 2001).

In constrained estimation, the parameter space over which optimization is performed is restricted to admissible values (e.g., variances are constrained to be positive; Schoenberg, 1997). To this end, inequality constraints that restrict parameter estimates to lower and upper bounds must be specified (see Savalei and Kolenikov, 2008). More specifically, in the constrained estimation approach, a multivalued function **h** is specified on the vector of SEM parameters, that is, **h**(θ) ≥ **0.** For example, if a parameter θ (e.g., correlation) is supposed to be bounded by a lower bound *l* and an upper bound *u*, that is, *l* ≤ θ ≤ *u*, the constraints would be given as follows: **h**(θ) = (θ − *l*, *u* − θ) ≥ (0, 0). Further possible constraints include restricting factor loadings or residual variances to positive values. Note that the constrained ML estimator ${\hat{\theta }}_{\text{CML}}$ is the parameter vector **θ** that maximizes the log-likelihood *l*(θ) in Equation 4 and fulfills the constraints that are imposed in **h**. Statistical inference can be based on the asymptotic covariance matrix that is obtained from plugging ${\hat{\theta }}_{\text{CML}}$ into the matrix of second derivatives of *l*(θ):

(6) $$\text{ACOV}\,\left({\hat{\theta }}_{\text{CML}}\right)={\left\{-{\left[\frac{\partial^{2}l\,\left(\theta \right)}{\partial \theta \,\partial \theta^{\prime }}\right]|}_{\theta ={\hat{\theta }}_{\text{CML}}}\right\}}^{-1},$$

where the diagonal elements of the *q* × *q* matrix are again used as estimates of standard errors (Dolan and Molenaar, 1991; but see Schoenberg, 1997, for alternative standard error estimation methods). The asymptotic covariance in Equation 6 can be enforced to be positive definite in empirical data if the parameter estimates are slightly pulled away from the boundary (e.g., by constraining correlations to the interval [−1+ε, 1−ε]).

In most SEM programs such as M*plus* (Muthén and Muthén, 2012) and lavaan (Rosseel, 2012), unconstrained ML estimation that does not impose any restrictions on the admissible parameter space is used as the default (see Kline, 2016). In the present article, we compare the performance of constrained and unconstrained ML estimation of CFA models with different Bayesian estimators. These are discussed in the next section.

### Bayesian Approach to Confirmatory Factor Analysis

In the Bayesian approach, statistical inference is based on the posterior distribution, which is determined by the likelihood function and the prior distribution π(θ) of model parameters (for a general introduction to the Bayesian approach, see Jackman, 2009; Gelman et al., 2014; van de Schoot et al., 2021). Using the observed data **X** (or the sufficient statistic **S**) and the prior distributions, the joint posterior distribution *p*(θ|**X)** of the parameters is determined by multiplying the likelihood with the prior:

(7) $$p\left(\theta |\text{X}\right)=\frac{L\,\left(\theta |\text{X}\right)\,\pi \,\left(\theta \right)}{\int L\,\left(\theta |\text{X}\right)\,\pi \,\left(\theta \right)\,\text{d}\,\theta }=L\left(\theta |\text{X}\right)\pi \left(\theta \right)C\propto L\left(\theta |\text{X}\right)\pi \left(\theta \right),$$

where *C* = 1/∫*L*(θ|**X**)π(θ)dθ is a normalizing constant. As can be seen, the posterior distribution is proportional to the product of the likelihood and the prior. If a researcher does not want to make assumptions about a parameter, non-informative (diffuse) prior distributions that are intended to have only a minimal influence on the results are selected (van de Schoot et al., 2021). Moreover, the Bayesian approach offers the opportunity to stabilize parameter estimates by specifying a weakly informative prior distribution π(θ) “which contains some information – enough to ‘regularize’ the posterior distribution, that is, to keep it roughly within reasonable bounds – but without attempting to fully capture one’s scientific knowledge about the underlying parameter” (Gelman et al., 2014, pp. 51). Thus, the idea is to incorporate a small amount of information into π(θ) that provides some direction for the estimation of model parameters but, at the same time, still allows the inferences to be driven by the likelihood (Baldwin and Fellingham, 2013; Chung et al., 2013; Lüdtke et al., 2013; Depaoli and Clifton, 2015). In the following, we discuss different Bayesian point estimates that are obtained from the posterior distribution *p*(θ|*X*).

#### Bayesian Point Estimates

Point estimates in the Bayesian approach are usually calculated by summarizing the center of the marginal posterior distribution of the particular parameters of interest (e.g., latent correlation). More formally, let θ_(__–_*_*d*_*_)_ = (θ_1_,…,θ*_*d*_*_–__1_, θ*_*d*_*_+__1_,…, θ*_*q*_*) denote the vector of parameters in which the *d*th entry of θ has been omitted. The univariate marginal posterior distribution of θ*_*d*_*, in which all other components of θ are integrated out, is given by:

(8) $$p_{d}\left(\theta_{d}|\text{X}\right)=\int p\left(\theta |\text{X}\right)\text{d}\theta_{\left(-d\right)}=C\int L\left(\theta |\text{X}\right)\pi \left(\theta \right)\text{d}\theta_{\left(-d\right)}.$$

Bayesian point estimates of θ*_*d*_* are obtained from location parameters (i.e., mean, median, mode) of the marginal posterior distribution. The posterior mean ${\hat{\theta }}_{d,\mathrm{EAP}}$ (*d* = 1,…,*q*) is given by the expectation of the posterior distribution:

(9) $${\hat{\theta }}_{d,\mathrm{EAP}}=\int \theta_{d}p_{d}\left(\theta_{d}|\text{X}\right)\text{d}\theta_{d}=\int \theta_{d}p\left(\theta |\text{X}\right)\text{d}\theta .$$

The posterior median (Med) ${\hat{\theta }}_{d,\mathrm{Med}}$ is the median of the marginal posterior distribution

(10) $$\int_{-\infty }^{{\hat{\theta }}_{d,\mathrm{Med}}}p_{d}\left(\theta_{d}|\text{X}\right)\text{d}\theta_{d}=0.5.$$

The posterior mode ${\hat{\theta }}_{d,\mathrm{MAP}}$ is given by the value that maximizes the marginal posterior distribution (maximum-a-posteriori; MAP):

(11) $${\hat{\theta }}_{d,\text{MAP}}=\underset{\theta_{d}}{\arg\,\max}p_{d}\,\left(\theta_{d}|\text{X}\right)$$

Note that all three Bayesian point estimates ${\hat{\theta }}_{d,\text{EAP}}$, ${\hat{\theta }}_{d,\text{Med}}$ and ${\hat{\theta }}_{d,\text{MAP}}$ are functionals of the joint posterior distribution and involve high-dimensional integration to obtain the marginal posterior distribution. In practice, simulation-based methods such as MCMC are often used to evaluate these high-dimensional integrals. As another option for a low number of parameters, numerical integration techniques can be employed (Held and Bové, 2014).

Alternatively, the mode of the joint posterior distribution *p*(θ|**X**) can be used as a Bayesian point estimate:

(12) $${\hat{\theta }}_{\text{PML}}=\underset{\theta }{\arg\,\max}p\,\left(\theta |\text{X}\right)=\underset{\theta }{\arg\,\max}\left[\log L\,\left(\theta |\text{X}\right)+\log\pi \,\left(\theta \right)\right].$$

Note that for the computation of ${\hat{\theta }}_{\text{PML}}$ (penalized maximum likelihood estimate; PML estimate; see Section “Penalized ML Estimation”) it is not required to evaluate the normalization constant of the posterior distribution. Three points need to be made about the mode of the joint posterior. First, with a diffuse prior (i.e., π is a constant function with respect to θ), the likelihood is proportional to the posterior distribution (see Equation 12), and the mode of the joint posterior coincides with the ML estimator. Second, it needs to be emphasized that the univariate modes ${\hat{\theta }}_{d,\mathrm{MAP}}$ (*d* = 1,…,*q*) of the marginal posterior distributions may not equal the components of the mode ${\hat{\theta }}_{\text{PML}}$ of the joint posterior distribution (Held and Bové, 2014). Note that the EAP has (in contrast to MAP and Med) the desirable property that it is invariant with respect to marginalization (see Equation 9); that is, the EAP for θ*_*d*_* of the univariate posterior distribution *p*_*d*_ equals the EAP of the multivariate posterior *p* (see Fox, 2010, p. 69). Third, for a multivariate normally distributed estimate ${\hat{\theta }}_{\text{ML}}∼\text{MVN}\,\left(\theta ,n^{-1}\,{\text{V}}_{1}\right)$ and a multivariate normal prior distribution (i.e., π(θ)≡MVN(θ_0_,**V**_0_)), it is well known that the posterior distribution is also multivariate normal (Gelman et al., 2014), that is

(13) $$p\left(\theta |\text{X}\right)\equiv \text{MVN}({\left({\text{V}}_{1}^{-1}+n^{-1}{\text{V}}_{0}^{-1}\right)}^{-1}\left({\text{V}}_{1}^{-1}{\hat{\theta }}_{\text{ML}}+n^{-1}{\text{V}}_{0}^{-1}\theta_{0}\right),n^{-1}{\left({\text{V}}_{1}^{-1}+n^{-1}{\text{V}}_{0}^{-1}\right)}^{-1}).$$

In this case, all estimators ${\hat{\theta }}_{\text{PML}}$, ${\hat{\theta }}_{d,\mathrm{MAP}}$, ${\hat{\theta }}_{d,\mathrm{Med}}$, and ${\hat{\theta }}_{d,\mathrm{EAP}}$ coincide. However, as ML estimates are only asymptotically normally distributed and often priors different from the normal distribution are used, it is not guaranteed that the different Bayesian estimators perform similarly, particularly in small samples. This fact is essential as different estimation methods produce different Bayesian point estimators. In the following, we distinguish between PML estimation and MCMC methods.

#### Penalized ML Estimation

Penalized ML estimation maximizes the log-posterior function:

(14) w⁢(θ)=l⁢(θ)+log⁢π⁢(θ).

The log-posterior is a function of the log-likelihood *l*(**θ**) = log *L*(**θ**|**X**) and additional information given by the prior log π(**θ**). The maximizer ${\hat{\theta }}_{\text{PML}}$ is also referred to as the maximum a posteriori (MAP) estimator (Gelman et al., 2014). Alternatively, the logarithm of the prior distribution logπ(θ) can be interpreted as a penalty term that is added to the log-likelihood function, which motivates the label “penalized” ML (Cole et al., 2014; see also Cousineau and Helie, 2013). It should also be emphasized that constrained ML estimation can be regarded as a variant of PML estimation when uniform prior distributions are imposed on the admissible parameter space (e.g., Rindskopf, 2012). In this case, it holds that ${\hat{\theta }}_{\text{PML}}={\hat{\theta }}_{\text{CML}}$.

Statistical inference in PML estimation can be obtained by plugging in the PML estimate ${\hat{\theta }}_{\text{PML}}$ into the matrix of second derivatives of the log-likelihood *l*(θ):

(15) $$\text{ACOV}\,\left({\hat{\theta }}_{\text{PML}}\right)={\left\{-{\left[\frac{\partial^{2}l\,\left(\theta \right)}{\partial \theta \,\partial \theta^{{}^{\prime }}}\right]|}_{\theta ={\hat{\theta }}_{\text{PML}}}\right\}}^{-1},$$

where the diagonal elements are used as estimates of standard errors. Note that the standard error estimates only rely on the log-likelihood *l*(θ) part and that the part of the prior distribution log π(θ) is ignored. The motivation for pursuing this strategy is that the prior is only used to stabilize the estimation. Based on experience from our simulations, it is vital to ignore the prior part in the computation of uncertainty for obtaining valid frequentist statistical inference because it would otherwise result in undercoverage.

Penalized maximum likelihood estimation has been shown to circumvent estimation problems, particularly when the likelihood is flat, and has been successfully applied to stabilize parameter estimates in a wide range of models such as logistic regression models (Firth, 1993; Heinze and Schemper, 2002), latent class models (Galindo-Garre and Vermunt, 2006; DeCarlo et al., 2011), item response theory models (Mislevy, 1986; Harwell and Baker, 1991), and multilevel models (Chung et al., 2013). It should also be emphasized that in the pre-MCMC era, PML estimation was the standard approach for obtaining estimates for Bayesian factor analysis models (e.g., Martin and McDonald, 1975; Press and Shigemasu, 1989) and Bayesian SEM models (e.g., Lee, 1981; Lee, 1992; Poon, 1999). Furthermore, PML estimation bears strong similarities to regularized ML estimation, in which, too, penalty functions are added to the log-likelihood (Jacobucci and Grimm, 2018; van Erp et al., 2019; Fan et al., 2020). Note that regularized estimation is often applied for effect selection, such as the determination of non-vanishing item loadings in factor analysis (Jin et al., 2018) or the allowance of non-invariant item parameters in multiple-group factor analysis (Huang, 2018).

#### MCMC Estimation

Another strategy that is used to obtain Bayesian estimates is to apply simulation-based techniques. This is motivated by the fact that in practice, the joint posterior distribution of the parameters is often difficult to evaluate because high-dimensional integration is required to compute the normalization constant *C* (see Equation 7**;** Held and Bové, 2014, Ch. 8). Simulation-based techniques – implemented in general-purpose Bayesian software such as WinBUGS (Spiegelhalter et al., 2003), JAGS (Plummer, 2003), NIMBLE (de Valpine et al., 2017), or Stan (Carpenter et al., 2017) – use MCMC algorithms to approximate the posterior distribution by iteratively sampling from conditional distributions. The most prominent MCMC methods are Gibbs sampling, Metropolis-Hastings sampling, and the no-U-turn sampler (Gelman et al., 2014; Junker et al., 2016).

In the present study, we implemented a Metropolis-Hastings step within a Gibbs sampling algorithm to estimate the parameters of the CFA model. The Metropolis-within-Gibbs algorithm uses the following sampling steps to generate observations from the conditional distributions. At the (*t* + 1)th iteration with current values $\left(\theta_{1}^{\left(t\right)},\ldots ,\theta_{q}^{\left(t\right)}\right)$ sample:

(16) $$\begin{array}{l} {\text{θ}}_{1}^{\left(t+1\right)}\;from\;p({\text{θ}}_{1}|X,{\text{θ}}_{2}^{\left(t\right)},{\text{θ}}_{3}^{\left(t\right)},\text{…},{\text{θ}}_{q}^{\left(t\right)})\\ {\text{θ}}_{2}^{\left(t+1\right)}\;from\;p({\text{θ}}_{2}|X,{\text{θ}}_{1}^{\left(t+1\right)},{\text{θ}}_{3}^{\left(t\right)},\text{…},{\text{θ}}_{q}^{\left(t\right)})\\ \vdots \\ {\text{θ}}_{q}^{\left(t+1\right)}\;from\;p({\text{θ}}_{q}|X,{\text{θ}}_{1}^{\left(t+1\right)},{\text{θ}}_{2}^{\left(t+1\right)},\text{…},{\text{θ}}_{q-1}^{\left(t+1\right)})[cpsbreak] \end{array}$$

There are *q* steps in the (*t* + 1)th iteration. All conditional distributions are unidimensional, and parameters are updated conditional on the latest value of the other parameters.

We now show how one component of the parameter vector θ, say θ*_*d*_*, is updated in the Metropolis-within-Gibbs algorithm. To generate a sample from the conditional distribution of θ*_*d*_* given the most recent values of the other parameters, we rewrite the conditional distribution using Bayes theorem:

(17) $$\begin{array}{l} p({\text{θ}}_{d}|X,{\text{θ}}_{1}^{\left(t+1\right)},{\text{θ}}_{2}^{\left(t+1\right)},\text{…},{\text{θ}}_{d-1}^{\left(t+1\right)},{\text{θ}}_{d+1}^{\left(t\right)},\text{…},{\text{θ}}_{q}^{\left(t\right)})\propto \\ L({\text{θ}}_{1}^{\left(t+1\right)},{\text{θ}}_{2}^{\left(t+1\right)},\text{…},{\text{θ}}_{d-1}^{\left(t+1\right)},{\text{θ}}_{d},{\text{θ}}_{d+1}^{\left(t\right)},\text{…},{\text{θ}}_{q}^{\left(t\right)}|X)\cdot \text{π}({\text{θ}}_{d}) \end{array}$$

The conditional distribution is proportional to the product of the likelihood and the prior distribution for θ*_*d*_*. A new value is sampled from a proposal distribution $\text{N}\,\left(\theta_{d}^{\left(t\right)},\tau_{\theta_{d}}^{2\,\left(t\right)}\right)$ where $\theta_{d}^{\left(t\right)}$ is the value of *θ_*d*_* from the previous iteration and $\tau_{\theta_{d}}^{2}$ is the proposal distribution standard deviation, which is adapted in the MCMC algorithm (see Section “Analysis Models and Outcomes”). Negative proposed values are not accepted, and the value from the previous iteration is used. Then the Metropolis-Hastings ratio is calculated as follows:

(18) $$\begin{array}{l} M({\text{θ}}_{d}^{\left(*\right)},{\text{θ}}_{d}^{\left(t\right)})\\ =\frac{L({\text{θ}}_{1}^{\left(t+1\right)},{\text{θ}}_{2}^{\left(t+1\right)},{\text{θ}}_{d-1}^{\left(t+1\right)},{\text{θ}}_{d}^{\left(*\right)},{\text{θ}}_{d+1}^{\left(t\right)},\text{…},{\text{θ}}_{q}^{\left(t\right)}|X)\cdot \text{π}({\text{θ}}_{d}^{\left(*\right)})}{L({\text{θ}}_{1}^{\left(t+1\right)},{\text{θ}}_{2}^{\left(t+1\right)},{\text{θ}}_{d-1}^{\left(t+1\right)},{\text{θ}}_{d}^{\left(t\right)},{\text{θ}}_{d+1}^{\left(t\right)},\text{…},{\text{θ}}_{q}^{\left(t\right)}|X)\cdot \text{π}({\text{θ}}_{d}^{\left(t\right)})}, \end{array}$$

where *M* = $M\,\left(\theta_{d}^{\left(*\right)},\theta_{d}^{\left(t\right)}\right)$ is the Metropolis-Hastings ratio as a function of the proposed value $\theta_{d}^{\left(*\right)}$ and the previous value $\theta_{d}^{\left(t\right)}$. The proposed value $\theta_{d}^{\left(*\right)}$ is then accepted and set to $\theta_{d}^{\left(t+1\right)}$with probability min(1, *M*). Acceptance rates of roughly between 0.40 and 0.50 are considered optimal in the literature (Hoff, 2009; Gelman et al., 2014) to obtain an MCMC chain that has relatively low autocorrelation and mixes well (i.e., moves around the sample space in a seemingly random fashion without any long-term trends).

When the chain converges, the draws $\theta^{\left(t\right)}=\left(\theta_{1}^{\left(t\right)},\ldots ,\theta_{d}^{\left(t\right)},\ldots ,\theta_{q}^{\left(t\right)}\right)$ can be seen as samples from the joint posterior distribution of the CFA model parameters (for a detailed discussion of assessing convergence in MCMC, see Cowles and Carlin, 1996; Gill, 2007; Jackman, 2009). Usually, the initial draws are discarded (burn-in phase) because the initial draws are affected by the starting values of the chain (Gelman et al., 2014). Bayesian point estimators are constructed from the samples of the posterior distribution. The expected a posteriori (EAP) estimator for a parameter *θ_*d*_*, ${\hat{\theta }}_{d,\text{EAP}}$, is obtained by averaging across the *T* iterations, that is,

(19) $${\hat{\theta }}_{d,\text{EAP}}=T^{-1}\,\sum_{t=1}^{T}\theta_{d}^{\left(t\right)}.$$

The median ${\hat{\theta }}_{d,\text{Med}}$ is estimated by computing the sample median of the draws $\theta_{d}^{\left(t\right)}$ (*t* = 1, …, *T*). The mode ${\hat{\theta }}_{d,\text{MAP}}$ can be defined as the univariate mode of the kernel density estimate (Silverman, 1998) of the univariate density for the sample of $\theta_{d}^{\left(t\right)}$ (*t* = 1, …, *T*) (see Johnson and Sinharay, 2016). Notably, it has been proposed that the multivariate mode (PML) could also be estimated by choosing the sampled parameter that maximizes the posterior distribution (see the discussion in the Stan users group^1^):

(20) $${\hat{\theta }}_{\mathrm{PML}-\mathrm{MCMC}}=\underset{t=1,\ldots ,T}{\arg\,\max}p\,\left(\theta^{\left(t\right)}|\text{X}\right).$$

However, if the PML is of primary interest, deterministic optimization using the Newton approach (see Section “MCMC Estimation” and Equation 12) is generally preferable.

The standard deviation of the posterior distribution can be used as a measure of uncertainty (Gelman et al., 2014). Comparable to a confidence interval in the frequentist approach, it is possible to calculate a Bayesian credibility interval (BCI). The BCI is based on percentile points of the posterior distribution and describes the probability that the interval covers the true value of the parameter after observing the data. Note that in contrast to the confidence interval in the frequentist approach, no assumptions about the sampling distribution (e.g., symmetry, normality) need to be made for the BCI.

Finally, it should also be emphasized that in the presented MCMC approach, the Bayesian point estimates are based on the marginal likelihood *L*(θ|**X**) – or *L*(θ|**S**) if the sufficient statistic **S** is used – in which the latent variables η are integrated out (Equation 4). However, in many applications of MCMC-based SEM, a joint estimation approach is used that relies on the joint likelihood *L*(θ,η|**X**), which also includes the latent variables η in the likelihood (Lee, 2007; Muthén and Asparouhov, 2012). In the joint estimation approach, the MCMC method^2^ generates samples for θ and η. When individual factor scores are not of interest, however, the latent variables η are nuisance parameters that can reduce the computational efficiency of the MCMC algorithm (Hayashi and Arav, 2006; Choi and Levy, 2017; Lüdtke et al., 2018; Hecht et al., 2020; Merkle et al., 2020).

#### Previous Research Comparing Bayesian Estimation Approaches

The different Bayesian point estimators, that is, ${\hat{\theta }}_{\text{PML}}$, ${\hat{\theta }}_{d,\text{EAP}}$, ${\hat{\theta }}_{d,\text{MAP}}$, and ${\hat{\theta }}_{d,\text{Med}}$, can be evaluated from a frequentist point of view – population parameters θ are treated as fixed but unknown constants, and the distribution of the Bayesian estimators is evaluated across all possible samples from the population (Stark, 2015). For simple univariate quantities (e.g., proportions, means), Bolstad and Curran (2017) compared frequentist properties (i.e., bias and RMSE) of mode, median, and mean using analytical derivations (see also Carlin and Louis, 2009; Efron, 2015). For more complex statistical models, several studies used simulated data to compare the performance of Bayesian estimators for different model parameters. Hoeschele and Tier (1995) compared the MAP and EAP (obtained from MCMC methods) for estimating variance components in multilevel models (see also Browne and Draper, 2006). Like the present study, Choi et al. (2011) evaluated two Bayesian estimators (MAP and EAP obtained from numerical integration) to estimate a polychoric correlation. The EAP estimates were biased and pulled toward the prior distribution (i.e., shrinkage effect), but less variable than the MAP estimates. In the context of IRT models, Azevedo et al. (2012; see also Azevedo and Andrade, 2013; Waller and Feuerstahler, 2017) and Kieftenbeld and Natesan (2012) compared PML and EAP estimates for a multiple-group 2PL model and the graded response model, respectively. In both studies, it turned out that the EAP estimates slightly outperformed PML in terms of RMSE (see also Bürkner, 2020). Yao (2014) and Johnson and Kuhn (2015) compared MAP and EAP estimation for person parameter estimation in unidimensional IRT models. Again, EAP estimates were biased (i.e., shrinkage effects) but were also less variable than MAP estimates (see also Johnson and Kuhn, 2015). For log-linear models, Galindo-Garre et al. (2004) found that the MAP outperformed the EAP for estimating main and interaction effects.

In the context of SEM and CFA models, systematic comparisons of the frequentist performance of Bayesian estimators are scarce. Simulation studies that evaluated the performance of different Bayesian estimators for estimating SEMs focused on either the Med (e.g., Hox et al., 2012, 2014; Depaoli and Clifton, 2015; Holtmann et al., 2016), the EAP (e.g., Lee and Song, 2004; Natesan, 2015) or the MAP (e.g., Zitzmann et al., 2016). One notable exception is the study by Miocevic et al. (2020) that evaluated the EAP, MAP, and Med for estimating an indirect effect (i.e., the product of two path coefficients) in a latent mediation model using MCMC methods. The relative performance of the different estimators in terms of RMSE depended on the specification of the prior distribution (accurate vs. inaccurate) and the size of the true indirect effect, with a slight disadvantage for the MAP when accurate priors were specified. However, it is unclear whether these findings generalize to other SEM parameters (e.g., loadings, latent correlations), making it difficult for applied researchers to choose between different Bayesian estimators. This lack of guidance is also reflected in the fact that popular software packages for SEM use different Bayesian estimators as default. The commercial software packages Mplus (Muthén, 2010) and Amos (Arbuckle, 2017) provide the Med, whereas the R package blavaan (Merkle and Rosseel, 2018) uses the EAP (see Taylor, 2019). In the present study, we evaluate the performance of four different Bayesian estimators for latent correlations and loadings in CFA models.

## CFA With Two Factors: Parameterization, Prior Distributions, and Estimation Methods

**FIGURE 1:**
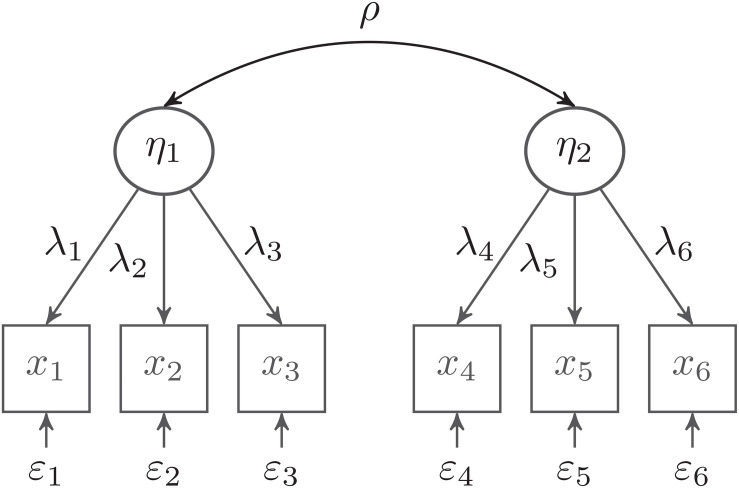
Confirmatory factor analysis (CFA) model with two latent factors. The variances of the latent factors are set to 1.0.

In the following, we consider a CFA model with two latent variables, which are each measured by three observed indicator variables (see Figure 1). We use this model to discuss three relevant issues in the practical application of the Bayesian approach: model parameterization, specification of prior distributions, and different Bayesian estimators (mode of the joint posterior, and mean, median, and mode of the marginal posterior). Although this is a very simple model, it is a building block for many more complicated SEM models, such as latent mediation models or multilevel SEMs (Hoyle, 2012; Kline, 2016).

### Parameterization

To mitigate estimation problems, it can be advantageous to choose a parameterization of the CFA model that transforms the optimization problem of estimating unbounded parameters into an optimization involving bounded parameters. We start with an unstandardized parameterization in which the two latent variables η_1_ and η_2_ are measured by indicators *x*_*j*_ (*j* = 1,…, 6):

(21) $$x_{j}=\lambda_{j}^{*}\,\eta_{\text{m}\,\left[j\right]}+\varepsilon_{j}^{*},$$

Where m[⋅] is a function that maps the item index *j* to the corresponding index m[*j*] of the latent variable, $\lambda_{j}^{*}$ are the unstandardized non-negative loadings, and $\varepsilon_{j}^{*}$ are normally distributed residuals with $\text{Var}\,\left(\varepsilon_{j}^{*}\right)=\omega_{j\,j}^{*}$. Two strategies for identifying the metric of the latent factors are often used (Kline, 2016; see also Gonzalez and Griffin, 2001). In the first strategy (reference variable method) the first loading of each latent factor is set to one, and the variances and covariance of the latent factors are freely estimated. In the second strategy, the variances of the latent variables are set to one, that is, Var(η_*1*_) = Var(η_2_) = 1, and the latent correlation between the factors is directly estimated. In the Bayesian framework, the reference variable method has been the most common choice (see Lee, 1981; Erosheva and Curtis, 2017; Merkle and Rosseel, 2018; Miocevic et al., 2020). This may be explained by the fact that the second strategy is not easily applicable to general SEM models because the variances of endogenous latent variables are not free parameters in standard SEM specifications (Kline, 2016; see also Kaplan and Depaoli, 2012; van Erp et al., 2018).

In this paper, we suggest a parameterization of the CFA model in which the parameters of interest are bounded, and the standardized loadings and the latent correlation are directly estimated (Little, 2013). Using a parameterization with bounded or standardized parameters has the advantage that it is straightforward to restrict correlations to admissible values between −1 and 1. This is more difficult to accomplish when the correlation is derived from the variances and covariance of the latent variables (e.g., very small variance estimates; Rindskopf, 1984)^3^. Furthermore, a parameterization with bounded parameters is often more convenient for applied researchers when specifying thoughtful prior distributions (Smid et al., 2020; Zitzmann et al., 2021). Let *σ_*j*_* denote the standard deviation of the observed indicator *x*_*j*_, then Equation 21 can be rewritten as:

(22) $$x_{j}=\sigma_{j}\,\left(\lambda_{j}\,\eta_{\text{m}\,\left[j\right]}+\varepsilon_{j}\right),$$

where *λ_*j*_* (*j* = 1,…,6) are the standardized loadings, and ε*_*j*_* are the residuals of the standardized solution. It can be shown that the parameterizations in Equations 21 and 22 are equivalent. It holds that $\sigma_{j}^{2}={\left(\lambda_{j}^{*}\right)}^{2}+\omega_{j\,j}^{*}$, $\lambda_{j}=\lambda_{j}^{*}/\sqrt{\omega_{j\,j}^{*}},$ and Var(ε_*j*_) = 1 – $\lambda_{j}^{2}$. Thus, the standardized error variance is positive if the standardized loadings are restricted to be positive. In many applications, especially with established scales, restricting loadings to positive values seems plausible because researchers commonly have strong presumptions on the direction of relationships between the observed and latent variables. Technically, the parameterization in Equation 22 can be implemented in the SEM framework by introducing an intermediate layer of latent variables (phantom variables; see Rindskopf, 1984) and non-linear constraints for the measurement error variances.^4^

In the present study, our main focus is on estimating the correlation between the latent variables, that is, Cov(η_1_, η_2_) = ρ. As we are interested in estimating the latent correlation that corrects for the unreliability of the scale scores, it is instructive to see how the reliability of the manifest sum score is related to the standardized loadings of the measurement model. In the data-generating model, we assume that the standardized loadings of the indicators for a latent factor are equal and set to λ (tau-congeneric measurement model; Traub, 1994). Then, the indicator-specific reliability is given by Rel_1_ = λ^2^, and the reliability of the sum score of *I* items is:

(23) $${\text{Rel}}_{I}=\frac{\lambda^{2}}{\lambda^{2}+\left(1-\lambda^{2}\right)/I}=\frac{{\text{Rel}}_{1}}{{\text{Rel}}_{1}+\left(1-{\text{Rel}}_{1}\right)/I}.$$

As can be seen, the reliability of the sum score Rel*_*I*_* is a function of the indicator-specific reliability Rel_1_ and the number of items. Thus, by rearranging terms, the reliability of an indicator can be written as:

(24) $${\text{Rel}}_{1}=\frac{{\text{Rel}}_{I}}{1+I\,\left(1-{\text{Rel}}_{I}\right)}.$$

For example, with *I* = 3, a standardized loading of λ = 0.58 translates into a reliability of 0.60 for the sum score (see Table 1). This relationship between the standardized loading and the reliability of the sum score is helpful when specifying the prior distributions for the loadings because, in most cases, it is easier to make plausible assumptions about the overall reliability of a scale than about every single item (Smid et al., 2020).

**TABLE 1:** Relationship between standardized loading, indicator-specific reliability, and reliability of sum score for three indicators.

λ	Rel_1_	Rel*_*I*_*
0.35	0.13	0.30
0.43	0.18	0.40
0.50	0.25	0.50
0.58	0.33	0.60
0.66	0.44	0.70
0.76	0.57	0.80
0.87	0.75	0.90

*λ = standardized loading; Rel_1_ = indicator-specific reliability; Rel*_*I*_* = reliability of sum score.*

### Specification of Prior Distributions

In the CFA model, the standardized loadings and latent correlations are bounded parameters. For bounded parameters, the beta distribution is a natural choice. The density *f* of the beta distribution *X* ∼ Beta(*a*, *b*) on the interval [0, 1] is given as:

(25) $$f\,\left(x\right)=B\,{\left(a,b\right)}^{-1}\,x^{a-1}\,{\left(1-x\right)}^{b-1},x\in \left[0,1\right]$$

where *B* is the Beta function. The mean and the variance can be computed as:

(26) $$\text{E}\,\left(X\right)=\frac{a}{a+b}\,\text{and}\,\text{Var}\,\left(X\right)=\frac{a\,b}{{\left(a+b\right)}^{2}\,\left(a+b+1\right)}.$$

Alternatively, the beta distribution can be parameterized as a function of a mean μ and a prior sample size ν, that is, *X* ∼ Beta(μ, ν), where μ = *a*(*a* + *b*)^–1^ and ν = *a* + *b* − 2 (Hoff, 2009). The prior sample size is explained by the fact that the uniform distribution, which reflects complete ignorance about a parameter, is given by setting *a* = *b* = 1. Thus, a prior sample size of ν = 1 + 1 − 2 = 0 corresponds to the uniform prior on [0, 1]. The variance of the beta distribution is given as Var(*X*) = μ(1 −μ)(ν + 3)^–1^. For the given μ and ν, the original *a* and *b* parameters are determined by *a* = (ν + 2)μ and *b* = (ν + 2)(1 −μ), respectively.

However, the beta distribution is only appropriate for parameters with a parameter space that equals [0, 1]. The four-parameter beta distribution (also known as a scaled, stretched, or generalized beta distribution) extends the support of the beta distribution to arbitrary bounded intervals and allows a more flexible specification of prior distributions (Johnson et al., 1994). The four-parameter beta distribution *Y* ∼ Beta4(*a*, *b*, *l*, *u*) can be obtained by shifting a beta-distributed random variable *X* ∼ Beta(*a*, *b*) by lower (*l*) and upper (*u*) bounds: *Y* = *l* + (*u* − *l*)*X*. The density of *Y* is then given as:

(27) $$f\,\left(x\right)={\left(u-l\right)}^{-1}\,B\,{\left(a,b\right)}^{-1}\,{\left(\frac{x-l}{u-l}\right)}^{a-1}\,{\left(\frac{u-x}{u-l}\right)}^{b-1},x\in \left[l,u\right]$$

Again, the four-parameter beta distribution can be reparameterized as *Y* ∼ Beta4(μ, ν, *l*, *u*) with a prior guess of μ = *a*(*a* + *b*)^–1^ and a prior sample size of ν = *a* + *b* − 2. The parameters of the original specification *Y* ∼ Beta4(*a*, *b*, *l*, *u*) can be obtained as *a* = (ν + 2)(μ− *l*)(*u* − *l*)^–1^ and *b* = (ν + 2)(*u* −μ)(*u* – *l*)^–1^. In previous research, the four-parameter beta distribution has been applied as prior distribution for item parameters in three-parameter logistic models (Zeng, 1997; Gao and Chen, 2005), and for correlations between observed scores (Gokhale and Press, 1982; O’Hagan et al., 2006) or latent variables in factor models (Muthén and Asparouhov, 2012; Merkle and Rosseel, 2018). However, to the best of our knowledge, we are not aware of any applications of the four-parameter beta distribution as prior distribution for loadings in the SEM framework (see Table 1 in van Erp et al., 2018, for an overview of prior distributions in the SEM context).

**FIGURE 2:**
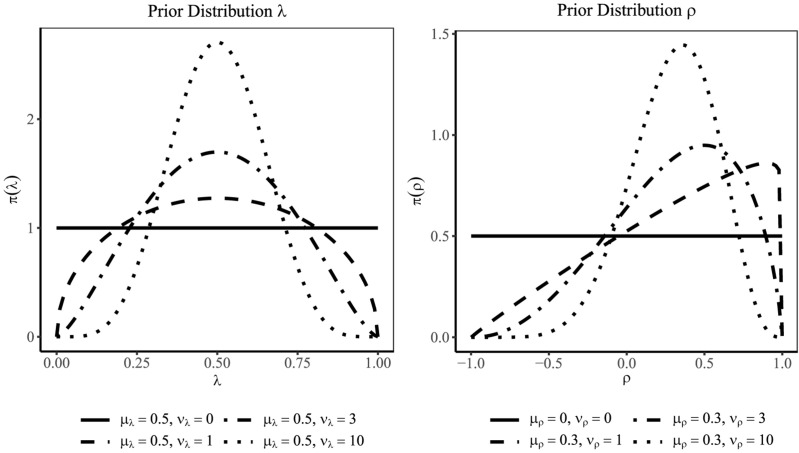
Four-parameter beta distributions for a standardized loading λ **(left panel)** and the correlation ρ **(right panel)**. With a larger prior sample size (ν_λ_ or ν_ρ_), the distribution puts more probability mass around the prior guess (μ_λ_ or μ_ρ_).

When specifying the lower and upper bounds of the four-parameter beta distribution, the numerical stability of parameter estimates can be improved if parameters are coerced further away from the boundaries by a small value ε (e.g., ε = 0.001). For a standardized loading λ that can be assumed to be bounded between 0 and 1, we suggest a Beta4(μ_λ_, ν_λ_, ε, 1 − ε) prior distribution and interpret μ_λ_ as a prior guess for the standardized loading and ν_λ_ as the sample size of prior observations on which the prior guess is based (Lüdtke et al., 2018)^5^. If only little information is available about the standardized loading or the reliability of a scale, a small value for ν_λ_ is chosen so that the prior distribution is only weakly centered around the prior guess μ_λ_. Figure 2 (left panel) shows for a prior guess of μ_λ_ = 0.50 (i.e., standardized loading of 0.50) how increasing ν_λ_ (i.e., prior sample sizes of 1, 3, and 10) changes the shape of the four-parameter beta distribution. Note that with μ_λ_ = 0.50 and ν_λ_ = 0, the four-parameter beta distribution corresponds to a uniform distribution on the interval [ε, 1 −ε], which reflects complete ignorance about the size of the loading.

For the latent correlation that is restricted to the interval [−1, 1], we suggest a Beta4(μ_ρ_, ν_ρ_, −1 + ε, 1 − ε) distribution where μ_ρ_ is the prior guess for the correlation and ν_ρ_ is again the prior sample size on which the prior guess is based (see for a similar approach Muthén and Asparouhov, 2012; Merkle and Rosseel, 2018). Figure 2 (right panel) illustrates the influence of the prior sample size ν_ρ_ (1, 3, and 10) on the shape of the Beta4(μ_ρ_, ν_ρ_, − 1 + ε, 1 − ε) with a prior guess of μ_ρ_ = 0.30. Setting μ_ρ_ = 0 and ν_ρ_ = 0 gives the uniform distribution on [−1 + ε, 1 − ε].

### Illustrative Comparison of Different Bayesian Point Estimates

To illustrate how the different Bayesian point estimates can produce different estimates of the latent correlation, we further simplify the two-factor model and assume that all loadings are equal. Thus, we need to estimate only two parameters^6^ : the correlation ρ (ranging between −1 and 1) and the standardized loading λ (ranging between 0 and 1). Thus, for this simplified model, the likelihood function L⁢(ρ,λ|S) is only a function of ρ and λ, given the sufficient statistic **S**. Furthermore, we assume uniform priors for both parameters (i.e., constant functions with respect to ρ and λ), which results in a joint posterior *p*(ρ,λ|**S**) that is proportional to the likelihood. In PML estimation, the mode of the joint posterior distribution is calculated as:

(28) $$\left({\hat{\rho }}_{\text{PML}},{\hat{\lambda }}_{\text{PML}}\right)=\underset{\left(\rho ,\lambda \right)}{\arg\,\max}p\,\left(\rho ,\lambda |\text{S}\right)=\underset{\left(\rho ,\lambda \right)}{\arg\,\max}L\,\left(\rho ,\lambda |\text{S}\right)$$

and ${\hat{\rho }}_{\text{PML}}$ is used as a point estimate of ρ. Note that this is the first component of the multivariate mode, which is calculated by directly maximizing the density of the joint posterior distribution. It becomes clear from Equation 28 that the PML estimate is also the constrained ML estimate because the likelihood function is maximized, that is ${\hat{\theta }}_{\text{PML}}={\hat{\theta }}_{\text{CML}}$.

In contrast, when using MCMC methods, the univariate mode (MAP), median (Med), and mean (EAP) are often used as point estimates for ρ. The marginal posterior distribution *p*_ρ_ of ρ is obtained by integrating the joint posterior *p*(ρ,λ|**S**) with respect to λ:

(29) $$p_{\rho }\left(\rho |\text{S}\right)=\int p\left(\rho ,\lambda |\text{S}\right)\text{d}\lambda =C\int L\left(\rho ,\lambda ,|\text{S}\right)\text{d}\lambda ,$$

where *C* = 1/∬*L*(ρ,λ|**S**)dρdλ is the normalizing constant. The corresponding marginal location parameters are given as follows:

(30) $${\hat{\rho }}_{\text{MAP}}=\underset{\rho }{\arg\,\max}p_{\rho }\left(\rho |\text{S}\right)=\underset{\rho }{\arg\,\max}\int L\left(\rho ,\lambda ,|\text{S}\right)\text{d}\lambda ,$$

(31) $$\int_{-\infty }^{{\hat{\rho }}_{\text{Med}}}p_{\rho }\,\left(\rho |\text{S}\right)\,\text{d}\,\rho =0.5,\text{and}$$

(32) $${\hat{\rho }}_{\text{EAP}}=\int \rho \,p_{\rho }\,\left(\rho |\text{S}\right)\,\text{d}\,\rho =\frac{\iint \rho \,L\,\left(\rho ,\lambda |\text{S}\right)\,\text{d}\,\rho \,\text{d}\,\lambda }{\iint L\,\left(\rho ,\lambda |\text{S}\right)\,\text{d}\,\rho \,\text{d}\,\lambda }.$$

In our simple bivariate case, the univariate EAP ${\hat{\rho }}_{\text{EAP}}$ can be calculated by numerically evaluating the posterior on a bivariate discrete grid of values ρ and λ. The integrals in Equation 32 can be obtained by numerical integration using a rectangle rule (see also Choi et al., 2011). Similarly, the median ${\hat{\rho }}_{\text{Med}}$ and the univariate MAP ${\hat{\rho }}_{\text{MAP}}$ can be obtained by a numerical evaluation of the integrals in Equations 30 and 31. However, this would not be practical with a larger number of parameters, and simulation-based MCMC techniques are needed to evaluate high-dimensional integrals (Held and Bové, 2014).

**FIGURE 3:**
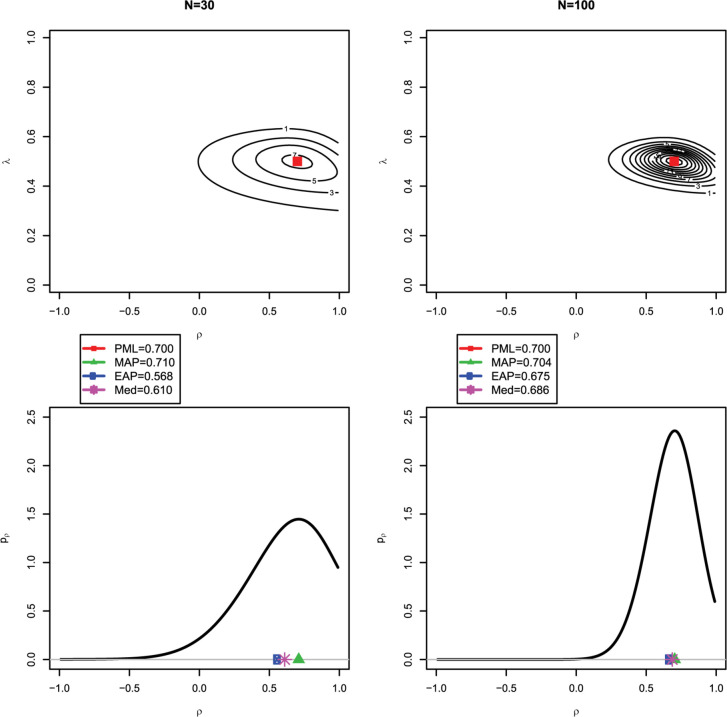
Illustrating the difference between the joint and marginal posterior distribution: the red square in the first row indicates the (multivariate) mode of the joint posterior distribution for *N* = 30 **(upper left panel)** and *N* = 100 **(upper right panel)**; the green triangle, blue circle, and purble star in the second row indicate the (univariate) mode, mean, and median of the marginal posterior distribution for the correlation ρ for *N* = 30 **(lower left panel)** and *N* = 100 **(lower right panel)**. Note that with *N* = 30, the mode of the joint posterior (PML) for ρ strongly deviates from the mean of the marginal posterior (EAP).

We now employ an idealized scenario to illustrate the difference between the different Bayesian estimates of the latent correlation. In this case, the empirical covariance matrix **S** (i.e., the sufficient statistic) obtained from the data was set to be equal to the true covariance matrix **Σ** = **Σ**(θ). Hence, the likelihood estimates (i.e., the constrained ML and the PML estimates) coincided with the data-generating parameters. The true correlation was ρ = 0.70, and the standardized loading was λ = 0.50. Figure 3 shows, for a small sample size of *N* = 30, a contour plot of the joint posterior distribution for ρ and λ (upper left panel) and the marginal posterior distribution of ρ (lower left panel). As can be seen, the mode of the joint posterior (${\hat{\rho }}_{\text{PML}}$ = 0.700) provides a different Bayesian estimate of the correlation than the mode (${\hat{\rho }}_{\text{MAP}}$ = 0.710), mean (${\hat{\rho }}_{\text{EAP}}$ = 0.568) or median (${\hat{\rho }}_{\text{Med}}$ = 0.610) of the marginal posterior. This can be explained by the fact that the marginal posterior is negatively skewed, and the mean and—to a slightly lesser extent—the median are pulled toward zero (i.e., shrinkage effect; see also Choi et al., 2011). However, with a larger sample of *N* = 100, the Bayesian estimates from the joint posterior (upper right panel) and the marginal posterior (lower right panel) agree more closely (${\hat{\rho }}_{\text{PML}}$ = 0.700, ${\hat{\rho }}_{\text{MAP}}$ = 0.704, ${\hat{\rho }}_{\text{EAP}}$ = 0.675, and ${\hat{\rho }}_{\text{Med}}$ = 0.686), and the marginal posterior distribution of ρ is more symmetrically centered around the true value of 0.70.

#### Illustrative Simulation Study

To further explore how these differences between the Bayesian point estimates affect their frequentist properties, we ran a small simulation study in which we manipulated the sample size (*N* = 30, 50, 100, and 1000) and the magnitude of the true correlation (ρ = 0.10, 0.30, 0.50, 0.70, and 0.90). The standardized loading was set to 0.50. We generated 1000 replications for each condition and calculated the bias, variability (i.e., the standard deviation of the empirical sampling distribution), and the RMSE (which combines bias and variability into a measure of accuracy) for the different point estimates (PML, MAP, EAP, and Med) of the latent correlation ρ.

The results are shown in Table 2 and confirm the findings from the illustration that the mode from the joint posterior (PML) and the mode from the marginal posterior (MAP) perform very similarly. Both produced approximately unbiased estimates of the latent correlation, except for the condition with a very large correlation (ρ = 0.90) and a small sample size (*N* = 30). By contrast, the mean (EAP) and the median (Med) of the marginal posterior provided negatively biased estimates, particularly in conditions with small sample sizes. However, the EAP and Med were also less variable (i.e., smaller empirical sampling variability) than the estimates produced by both the PML estimate and the MAP, resulting in overall more accurate estimates in terms of the RMSE, which combines bias and variability. The results also show that there is a turning point at which, with a larger true correlation, the bias introduced by the EAP outweighs the gains in efficiency (i.e., less variable estimates of the EAP). Thus, the EAP seems to be most beneficial with a small to moderate true correlation (i.e., ρ ≤ 0.50) and does not generally result in more accurate estimates of the latent correlation. A very similar pattern holds true for the Med. However, in almost all conditions, the Med was outperformed by either the EAP or the MAP in terms of RMSE. Notably, the multivariate mode (PML) performed similarly to the univariate mode (MAP). However, in other models, particularly with strongly correlated parameter estimates, the multivariate and univariate modes can provide substantially different point estimates.^7^ Finally, the findings confirm that with large samples, the different Bayesian point estimates converge and produce almost identical results.

**TABLE 2:** Illustrating differences between the mode of the joint posterior (PML) and the mode (MAP), median (Med), and mean (EAP) of the marginal posterior as point estimators of the correlation: bias, standard deviation, and RMSE as a function of the true correlation (ρ) and sample size (N).

		Bias				SD				RMSE			
ρ	*N*	PML	MAP	Med	EAP	PML	MAP	Med	EAP	PML	MAP	Med	EAP
0.1	30	0.000	0.001	–0.017	–0.024	0.417	0.416	0.322	0.293	0.417	0.416	0.322	0.294
	50	0.005	0.007	–0.003	–0.007	0.301	0.305	0.273	0.259	0.301	0.305	0.272	0.259
	100	–0.011	–0.009	–0.011	–0.012	0.210	0.213	0.208	0.205	0.211	0.213	0.208	0.206
	1000	0.001	0.001	0.001	0.001	0.061	0.061	0.060	0.060	0.061	0.061	0.060	0.060
0.3	30	–0.001	0.002	**−0.053**	**−0.073**	0.410	0.406	0.308	0.281	0.410	0.405	0.312	0.290
	50	0.007	0.010	–0.019	–0.031	0.294	0.295	0.258	0.246	0.294	0.295	0.259	0.248
	100	0.002	0.006	–0.002	–0.006	0.196	0.198	0.192	0.189	0.196	0.198	0.192	0.189
	1000	–0.002	–0.002	–0.003	–0.003	0.061	0.061	0.061	0.061	0.061	0.061	0.061	0.061
0.5	30	0.005	0.006	**−0.093**	**−0.126**	0.345	0.340	0.262	0.244	0.345	0.340	0.278	0.274
	50	0.006	0.010	–0.040	**−0.060**	0.278	0.276	0.237	0.226	0.278	0.276	0.241	0.234
	100	0.004	0.008	–0.008	–0.015	0.186	0.185	0.174	0.169	0.186	0.185	0.174	0.170
	1000	0.001	0.002	0.001	0.001	0.060	0.060	0.060	0.060	0.060	0.060	0.060	0.060
0.7	30	–0.026	–0.024	**−0.150**	**−0.192**	0.299	0.292	0.231	0.221	0.300	0.293	0.275	0.292
	50	–0.004	–0.002	**−0.081**	**−0.108**	0.234	0.229	0.190	0.183	0.234	0.229	0.206	0.212
	100	0.001	0.003	–0.030	–0.043	0.169	0.166	0.142	0.137	0.169	0.166	0.145	0.143
	1000	–0.001	0.000	–0.001	–0.001	0.054	0.054	0.055	0.055	0.054	0.054	0.055	0.055
0.9	30	**−0.069**	**−0.070**	**−0.227**	**−0.277**	0.218	0.213	0.182	0.181	0.228	0.224	0.290	0.331
	50	–0.039	–0.039	**−0.145**	**−0.178**	0.164	0.160	0.132	0.131	0.169	0.165	0.196	0.221
	100	–0.021	–0.022	**−0.083**	**−0.102**	0.124	0.122	0.095	0.091	0.126	0.124	0.126	0.137
	1000	0.001	0.001	–0.006	–0.009	0.052	0.052	0.045	0.043	0.052	0.052	0.045	0.044

**SD* = standard deviation of empirical sampling distribution; RMSE = root mean square error; ρ = true latent correlation; *N* = sample size; PML = mode of joint posterior (obtained from maximizing the joint posterior); MAP = mode of marginal posterior (obtained from maximizing the marginal posterior); Med = median of marginal posterior (obtained from numerical integration); EAP = mean of marginal posterior (obtained from numerical integration). Biases smaller than −0.05 or larger than 0.05 are printed in bold.*

We also investigated the performance of the different Bayesian point estimates for the loading parameter λ (see for the detailed results Supplementary 1 at https://doi.org/fwr7). Across all conditions (i.e., true correlations and sample sizes) the biases for the four estimators were relatively small (PML: *M* = −0.001, range = −0.010 to 0.005; MAP: *M* = −0.004, range = −0.019 to 0.006; Med: *M* = −0.013, range = −0.040 to 0.003; EAP: *M* = −0.017, range = −0.050 to 0.001). In addition, the PML provided slightly more accurate estimates in terms of RMSE than the three Bayesian estimates that were based on the marginal posterior.

In the following, we report the results of two simulation studies that provide a more comprehensive comparison of the different Bayesian point estimates. In these simulations, MCMC methods are used to evaluate the high-dimensional integrals that are needed for computing the MAP, EAP, and Med from the marginal posterior distribution.

## Simulation Study 1

Simulation study 1 had two main goals. First, we evaluated the performance of the different Bayesian estimators and compared them with unconstrained ML estimation. For small sample sizes, we expected unconstrained ML estimation to show serious estimation problems (i.e., non-convergence or inadmissible parameter estimates). In addition, based on our illustration, we assumed that using the EAP (obtained from MCMC) as a point estimate would produce more stable estimates of latent correlations than the multivariate mode from PML estimation, particularly in conditions with small sample sizes and small to moderate correlations. Second, we evaluated the extent to which the parameter estimates of the Bayesian approach are sensitive to different specifications of the prior distributions for the standardized loadings and the latent correlation. We assumed that by choosing weakly informative and correctly specified prior distributions (i.e., four-parameter beta distributions), the estimates of the latent correlations could be stabilized. Furthermore, we explored whether the Bayesian approach produces more accurate estimates, even with mildly misspecified prior distributions. Overall, we expected the impact of choosing different prior distributions to be more pronounced with small sample sizes.

### Simulation Model and Conditions

The data-generating model was a two-factor CFA model, as given by Figure 1. Each factor was measured by three mean-centered and normally distributed indicators. The indicators were assumed to be parallel, with standardized loadings of 0.50 and a variance of one. This resulted in a reliability of Rel*_*I*_* = 0.50 for each scale (i.e., sum score of the three items) and a reliability of Rel_1_ = 0.25 for a single indicator. We manipulated the latent correlation between the two factors (ρ = 0.30, 0.50, and 0.70) and the sample size (*N* = 30, 50, and 100). For each of the 3 × 3 = 9 conditions, we generated 1,000 simulated data sets.

### Analysis Models and Outcomes

Each of the simulated data sets was analyzed with a two-factor CFA model in which the loadings were freely estimated, and the variances of the two factors were each fixed to one. The model had 21 − 13 = 8 degrees of freedom (the mean structure was assumed to be saturated). Two ML estimation approaches were used. In unconstrained ML estimation, we imposed no constraints on the parameter estimates (loadings, residual variances, and the latent correlation). In constrained ML estimation, we used the parameterization in which standard deviations of the indicators are constrained to be positive, the standardized loadings are restricted to the interval [0, 1], and the latent correlation is restricted to the interval [−1, 1]^8^. In the Bayesian approach, we used PML estimation and MCMC methods, and varied the prior distribution for the standardized loadings and the latent correlation. PML estimation was implemented using a quasi-Newton optimization (employing the nlminb optimizer in the R package stats) using the wrapper function pmle from the R package LAM (Robitzsch, 2020). The standard errors were calculated based on the second derivatives of the observed log-likelihood function (see Equation 15). The estimated standard errors were used to calculate 95% confidence intervals.

For the MCMC method, we implemented an adaptive Metropolis-Hastings algorithm in which the proposal distribution is adapted during the burn-in phase (see Equation 18; Draper, 2008). In this procedure, a desirable acceptance rate *r* along with a tolerance level (*r* − δ, *r* + δ) is specified (in our case *r* = 0.45 and δ = 0.10). Then, in the burn-in phase of the algorithm, the empirical acceptance rates *r*^∗^ for each parameter are calculated in batches of 50 iterations. At the end of each batch, the proposal distribution standard deviation (e.g., for the latent correlation τ_ρ_) is updated as follows:

(33) $$\tau_{\rho }=\left\{ \begin{array}{c} {\tilde{\tau }}_{\rho }\,\left(2-\frac{\left(1-r^{*}\right)}{1-r}\right)\,\text{if}\,r^{*}>r+\delta \\ {\tilde{\tau }}_{\rho }\,\left(1/\left(2-\frac{r^{*}}{r}\right)\right)\,\text{if}\,r^{*}<r-\delta \\ {\tilde{\tau }}_{\rho }\,\text{else} \end{array}\right.,$$

Where *r*^∗^ is the empirical acceptance rate from the most recent batch of iterations, and ${\tilde{\tau }}_{\rho }$ is the proposal distribution standard deviation that was used in the most recent batch. Thus, the proposal distribution standard deviations are modified if the acceptance rate is not within the tolerance level (*r* − δ, *r* + δ). The modification of the proposal distributions was stopped after the burn-in phase (2,500 iterations). To evaluate the tuning phase for the proposal distribution standard deviations, we investigated the empirical acceptance rates for one condition of the main simulation. The average acceptance rate for the model parameters was close to the desired value of 0.45, which is considered optimal in the literature to achieve efficient MCMC chains (Hoff, 2009).

This algorithm was implemented using the function amh from the R package LAM (Robitzsch, 2020). Before running the main simulation study, we investigated the behavior of the MCMC sampler in preliminary simulations by inspecting two criteria: (a) the potential scale reduction factor (PSR; Gelman et al., 2014), and (b) the effective sample size (see Zitzmann and Hecht, 2019, for a discussion). Applying these two criteria suggested that an average chain length of 5,000 iterations with a burn-in period of 2,500 iterations was sufficient to provide a good approximation of the posterior distribution. The Bayesian point estimates were defined as the mean (EAP), mode (MAP), and median (Med) of the marginal posterior distribution. Furthermore, the Bayesian credible interval (BCI) was defined by the 2.5^*th*^ and the 97.5^*th*^ percentiles of the posterior distribution (Gelman et al., 2014).

For both the PML and the MCMC methods, we varied the prior distributions for the standardized loadings and the latent correlation. For each standardized loading, we specified a four-parameter beta distribution Beta4(μ_λ_, ν_λ_, ε, 1 −ε) with a prior guess of μ_λ_ = 0.5 and prior sample sizes of ν_λ_ = 1 and ν_λ_ = 3 (see Figure 2). In addition, we included a prior distribution with μ_λ_ = 0.5 and ν_λ_ = 0, which corresponds to a uniform distribution on [ε, 1 −ε]. For the latent correlation, we specified a four-parameter beta distribution Beta4(μ_ρ_, ν_ρ_, −1 + ε, 1 −ε) with a prior guess that matched the true correlation of the data-generating model (i.e., μ_ρ_ = ρ) and two levels of prior sample sizes (ν_ρ_ = 1 and 3). We also specified a prior distribution with μ_ρ_ = 0 and ν_ρ_ = 0, which corresponds to a uniform distribution on [−1 + ε, 1 −ε]. This resulted in 3 (loadings) × 3 (correlations) = 9 different specifications of the prior distributions. Note that these prior distributions were correctly specified (i.e., prior guess matched the true population value or uniform prior distribution was specified) and only differed in the amount of information that was incorporated into the prior specification (i.e., prior sample size).

We also investigated the impact of misspecified prior distributions. To this end, we specified a wide range of four-parameter beta distributions for the standardized loadings and the correlation. For the standardized loadings, we included misspecified priors with a prior guess of μ_λ_ = 0.80 and prior sample sizes of ν_λ_ = 1 and ν_λ_ = 3. For the latent correlation, we specified a prior distribution with a prior guess of μ_ρ_ = 0.50 and prior sample sizes of ν_ρ_ = 1, and ν_ρ_ = 3. However, we also included misspecified priors that underestimated (with a prior guess of μ_ρ_ = 0.20) or overestimated (with a prior guess of μ_ρ_ = 0.80) the true correlation. Again, each misspecified prior was included with prior sample sizes of ν_ρ_ = 1 and ν_ρ_ = 3. In addition, we used an uniform distribution for the latent correlation (i.e., μ_ρ_ = 0 and ν_ρ_ = 0). These prior settings for correlations were fully crossed with the different prior settings for correctly and misspecified prior settings on standardized factor loadings. In total, we specified 5 (standardized loadings) × 7 (correlations) = 35 models with different prior specifications, and we estimated them with both the PML and the MCMC methods.

For the standard deviations of the indicator variables, we used improper prior distributions that are constant (Muthén and Asparouhov, 2012). The specification of the improper prior distribution for the standard deviation was held constant across the conditions of the simulation and the analysis models. The R code for the data-generating model and the different analysis models is provided in Supplementary 2 at https://doi.org/fwr7.

We used three criteria to evaluate the different estimation approaches: bias, RMSE, and coverage rate. Bias was calculated by determining the difference between the mean parameter estimate and the true population parameter value from each design cell. We assessed the overall accuracy with the (empirical) RMSE, which combines the squared empirical bias and the variance of the parameter estimates into a measure of overall accuracy. Finally, we determined the coverage rate of the 95% confidence intervals. A coverage rate between 91% and 98% was considered acceptable (Muthén and Muthén, 2002).

### Results

We first report the results for the two ML estimation approaches. Second, we compare the different Bayesian estimators in the case of correctly specified prior distributions. Third, we investigate the impact of misspecified prior distributions on the performance of the Bayesian approach.

#### ML Estimation

For unconstrained ML estimation, a solution was considered to show estimation problems when the algorithm did not converge using the defaults in the nlminb optimizer or when the algorithm converged to a solution that included an inadmissible estimate (i.e., correlation smaller than −1 or larger than 1). Table 3 shows that the percentage of estimation problems for unconstrained ML estimation strongly depended on the sample size and the magnitude of the true correlation. For example, with *N* = 30 and ρ = 0.30, only 54.5% of the replications converged, and 51.3% of the replications provided converged solutions with admissible estimates. In contrast, for constrained ML estimation, all replications converged. However, in small samples and with a large correlation, a substantial percentage of the solutions for constrained ML estimation showed values at the boundary of the parameter space (e.g., estimated correlation equals one). Furthermore, the results also show that with increasing sample size, the estimates of unconstrained ML and constrained ML converged to each other. For example, in the condition with *N* = 100 and a large correlation (ρ = 0.70), unconstrained and constrained estimation provided (numerically) identical estimates of the latent correlation in 91.6% of the replications.

**TABLE 3:** Simulation study 1: percentage of solutions with estimation problems for unconstrained maximum likelihood (ML) estimation and constrained maximum likelihood (CML) estimation by magnitude of the true correlation (ρ) and sample size (N).

	ML Conv			ML Conv+Adm			CML Boundary			ML = CML		
	ρ			ρ			ρ			ρ		
*N*	0.3	0.5	0.7	0.3	0.5	0.7	0.3	0.5	0.7	0.3	0.5	0.7
30	54.5	64.4	67.9	51.3	57.3	53.4	6.3	11.1	21.6	28.0	33.0	34.8
50	73.1	82.7	88.0	70.9	77.5	73.9	3.4	6.3	17.0	53.1	61.7	64.3
100	94.0	97.6	99.6	93.9	96.4	92.8	0.4	1.4	7.2	87.1	92.8	91.6

*ML Conv = percentage of converged solutions for unconstrained ML estimation; ML Conv+Adm = percentage of converged solutions with admissible values for unconstrained ML estimation, that is, estimated correlation was within the interval [−1, 1]; CML Boundary = percentage of solutions for constrained ML estimation with boundary estimate for correlation; ML = CML = percentage of solutions in which estimated correlation for ML and CML estimations were numerically identical (up to three decimal places). Note that ML Conv+Adm are not conditional percentages and, thus, values cannot exceed those of ML Conv.*

Table 4 shows the bias and RMSE for unconstrained and constrained ML estimation as a function of the sample size and the true correlation. The results are presented for three different subsets of replications. First, we included only replications in which unconstrained ML estimation converged and estimated correlations had admissible values; that is, they fell within the range of −1 and 1 (“Conv+Adm” in Table 4). Second, we present results for all replications in which unconstrained ML estimation converged, and inadmissible values (i.e., correlations smaller than −1 or larger than 1) were truncated to −1 or 1 (“Conv”). Third, we show the results for all replications (“All”). Note that only constrained ML estimation converged for all replications. As can be seen, the two approaches performed very similarly across the different subsets of replications. The results also show that the estimates produced from replications without estimation problems (“Conv+Adm”) are a highly selective set of estimates that strongly differ in terms of RMSE from the estimates that are provided by the full set of replications (“All”). In the following, we use constrained ML estimation, which is equivalent to PML estimation with uniform distributions on the admissible parameter space, and compare it with the Bayesian estimation approach.

**TABLE 4:** Simulation study 1: bias and RMSE for unconstrained maximum likelihood (ML) estimation and constrained maximum likelihood (CML) estimation of the latent correlation as a function of the true correlation (ρ), the sample size (*N*), and different sets of replications.

		Bias					RMSE				
		Conv+Adm.		Conv.		All	Conv+Adm.		Conv.		All
ρ	*N*	ML	CML	ML	CML	CML	ML	CML	ML	CML	CML
0.3	30	0.022	**0.055**	0.043	**0.068**	–0.016	0.352	0.358	0.395	0.395	0.411
	50	0.025	0.035	0.040	0.049	–0.003	0.284	0.288	0.310	0.309	0.328
	100	0.003	0.006	0.004	0.006	–0.004	0.202	0.201	0.203	0.203	0.210
0.5	30	–0.037	–0.003	0.019	0.048	–0.025	0.299	0.295	0.332	0.322	0.360
	50	–0.042	–0.032	–0.015	–0.005	–0.049	0.266	0.262	0.299	0.285	0.316
	100	0.000	0.002	0.006	0.008	0.003	0.196	0.194	0.202	0.201	0.206
0.7	30	−**0.124**	−**0.088**	–0.034	–0.008	−**0.068**	0.299	0.269	0.299	0.274	0.344
	50	−**0.071**	−**0.063**	–0.012	–0.005	–0.035	0.242	0.234	0.252	0.245	0.275
	100	–0.026	–0.025	–0.004	–0.003	–0.004	0.172	0.171	0.184	0.183	0.185

*RMSE = root mean square error; ρ = true latent correlation; *N* = sample size; ML = unconstrained ML estimation; CML = constrained ML estimation. Conv+Adm = all replications in which unconstrained ML converged with admissible values, that is, estimated correlation was within the interval [−1, 1]; Conv = all replications in which unconstrained ML estimation converged and estimated correlations outside [−1, 1] are truncated to −1 or 1; All = all replications (only constrained ML produced estimates for all replications). Biases smaller than −0.05 or larger than.05 are printed in bold.*

#### Bayesian Estimation With Correctly Specified Priors

Table 5 shows the bias for PML and the EAP (obtained from the MCMC method) with uniform and different correctly specified prior distributions as a function of the sample size and the magnitude of the true correlation. In these specifications, the prior guesses for the correlation (i.e., μ_ρ_ = 0.30, 0.50, or 0.70) as well as the standardized loading (i.e., μ_λ_ = .50) were set to the true value (when the prior sample sizes of the corresponding priors were at least one). Note that when using a prior sample size of zero, μ_ρ_ was set to zero, and a uniform distribution was specified. As can be seen, both the PML and the EAP produced biased estimates of the correlation, particularly when the sample sizes were small (*N* ≤ 50). In addition, there was a tendency that increasing the prior sample size from ν_ρ_ = 1 to ν_ρ_ = 3, and thereby selecting a more informative prior distribution for the latent correlation, decreased the bias for both the PML (ν_ρ_ = 1: *M* = 0.129, *SD* = 0.053, range = 0.021 to 0.213; ν_ρ_ = 3: *M* = 0.099, *SD* = 0.044, range = 0.016 to 0.210) and the EAP (ν_ρ_ = 1: *M* = 0.086, *SD* = 0.038, range = −0.006 to 0.164; ν_ρ_ = 3: *M* = 0.036, *SD* = 0.023, range = −0.023 to 0.122). Interestingly, the results were less clear for increasing the sample size from ν_ρ_ = 0 to ν_ρ_ = 1, particularly for the PML.

**TABLE 5:** Simulation study 1: bias and RMSE for the mode of the joint posterior (PML) and the mean of the marginal posterior (EAP) as Bayesian point estimates of the latent correlation with different correctly specified prior distributions as a function of the sample size and true correlation (ρ).

				Bias			SD			RMSE Gain		
				ρ			ρ			ρ		
*N*	*Meth*	ν_ρ_	*ν*_λ_	0.3	0.5	0.7	0.3	0.5	0.7	0.3	0.5	0.7
30	PML	0	0	–0.001	−**0.052**	−**0.069**	0.415	0.393	0.330	100	100	100
		0	1	0.034	–0.010	–0.017	0.434	0.399	0.313	105	101	93
		0	3	0.043	0.001	–0.003	0.424	0.389	0.302	103	98	90
		1	0	**0.086**	**0.153**	**0.158**	0.370	0.364	0.264	92	100	91
		1	1	**0.119**	**0.198**	**0.192**	0.374	0.347	0.230	95	101	89
		1	3	**0.121**	**0.213**	**0.209**	0.364	0.346	0.219	92	102	90
		3	0	0.049	**0.092**	**0.165**	0.283	0.257	0.233	69	69	85
		3	1	**0.086**	**0.117**	**0.190**	0.291	0.249	0.208	73	69	83
		3	3	**0.088**	**0.121**	**0.210**	0.280	0.245	0.196	71	69	85
	EAP	0	0	−**0.072**	−**0.146**	−**0.215**	0.284	0.263	0.236	71	76	95
		0	1	–0.021	−**0.077**	−**0.124**	0.331	0.300	0.245	80	78	81
		0	3	–0.008	−**0.057**	−**0.098**	0.342	0.310	0.248	83	79	79
		1	0	**0.058**	**0.129**	**0.104**	0.221	0.173	0.149	55	54	54
		1	1	**0.081**	**0.153**	**0.133**	0.266	0.209	0.137	67	65	57
		1	3	**0.089**	**0.164**	**0.136**	0.277	0.221	0.138	70	69	58
		3	0	–0.006	0.002	**0.087**	0.169	0.151	0.105	41	38	41
		3	1	0.018	0.035	**0.112**	0.191	0.178	0.116	46	46	48
		3	3	0.024	0.045	**0.122**	0.197	0.185	0.118	48	48	50
50	PML	0	0	–0.018	–0.010	–0.041	0.335	0.302	0.275	100	100	100
		0	1	0.004	0.021	–0.011	0.343	0.293	0.255	102	97	92
		0	3	0.006	0.027	0.001	0.331	0.286	0.247	99	95	89
		1	0	0.042	**0.139**	**0.148**	0.312	0.321	0.248	94	116	104
		1	1	**0.061**	**0.165**	**0.167**	0.310	0.309	0.228	94	116	102
		1	3	**0.060**	**0.170**	**0.188**	0.301	0.305	0.216	91	116	103
		3	0	0.021	**0.070**	**0.149**	0.250	0.234	0.228	75	81	98
		3	1	0.045	**0.094**	**0.161**	0.257	0.220	0.212	78	79	96
		3	3	0.045	**0.097**	**0.181**	0.249	0.217	0.202	75	79	98
	EAP	0	0	−**0.068**	−**0.091**	−**0.147**	0.251	0.230	0.213	77	82	93
		0	1	–0.033	–0.037	−**0.085**	0.278	0.245	0.213	83	82	82
		0	3	–0.026	–0.022	−**0.065**	0.283	0.250	0.215	85	83	81
		1	0	0.013	**0.103**	**0.095**	0.214	0.191	0.149	64	72	63
		1	1	0.027	**0.123**	**0.117**	0.243	0.217	0.141	73	82	66
		1	3	0.032	**0.131**	**0.127**	0.250	0.226	0.142	75	86	68
		3	0	–0.023	–0.004	**0.074**	0.170	0.158	0.131	51	52	54
		3	1	–0.004	0.027	**0.096**	0.191	0.175	0.138	57	59	60
		3	3	0.003	0.036	**0.102**	0.195	0.180	0.138	58	61	62
100	PML	0	0	–0.006	–0.004	0.005	0.222	0.211	0.183	100	100	100
		0	1	0.005	0.008	0.013	0.220	0.203	0.176	99	96	97
		0	3	0.009	0.012	0.017	0.216	0.199	0.173	97	95	95
		1	0	0.021	**0.099**	**0.165**	0.216	0.266	0.196	98	134	140
		1	1	0.032	**0.101**	**0.166**	0.211	0.252	0.191	96	129	139
		1	3	0.035	**0.100**	**0.168**	0.208	0.246	0.189	95	126	139
		3	0	0.016	0.040	**0.156**	0.193	0.185	0.187	87	89	134
		3	1	0.027	0.048	**0.161**	0.190	0.179	0.184	86	88	134
		3	3	0.030	**0.052**	**0.159**	0.188	0.177	0.181	85	87	132
	EAP	0	0	–0.039	−**0.052**	−**0.056**	0.191	0.188	0.166	88	92	96
		0	1	–0.019	–0.028	–0.033	0.198	0.190	0.161	90	91	90
		0	3	–0.014	–0.018	–0.022	0.200	0.189	0.160	90	90	88
		1	0	–0.006	0.045	0.105	0.183	0.199	0.136	82	96	94
		1	1	0.007	**0.059**	**0.114**	0.190	0.202	0.132	86	100	96
		1	3	0.013	**0.064**	**0.119**	0.192	0.203	0.131	87	101	97
		3	0	–0.020	–0.011	**0.074**	0.157	0.158	0.138	71	75	86
		3	1	–0.004	0.007	**0.085**	0.164	0.159	0.136	74	75	88
		3	3	0.002	0.015	**0.089**	0.167	0.159	0.135	75	76	88

**SD* = standard deviation of empirical sampling distribution; RMSE = root mean square error. PML = penalized maximum likelihood (mode of joint posterior); EAP = mean of marginal posterior; ν_ρ_ = prior sample size for latent correlation; ν_λ_ = prior sample size for standardized loading; biases smaller than -0.05 or larger than 0.05 are printed in bold. For the RMSE gain, the RMSE of PML estimation with uniform prior distributions is used as a reference method; values smaller than 100 indicate that the RMSE of the respective method is lower than the RMSE of the reference method.*

For the RMSE, which combines bias and the variability of an estimator, we used the PML method with uniform prior distributions on the standardized loadings and the latent correlation as a reference method. As this specification of the PML method is equivalent to constrained ML estimation, it allows a direct comparison of the Bayesian approaches with the best performing ML approach. The RMSE gain in Table 5 reports the relative gain of an estimator compared to the reference method (i.e., values larger/smaller than 100 indicate that the RMSE for the respective method is larger/smaller than for the reference method). The results show that the EAP obtained from the MCMC method clearly outperformed PML estimation across all sample size conditions and true values of the latent correlation. As expected from the illustration, the differences between the mode of the joint posterior distribution (PML) and the mean (EAP) of the marginal posterior were most pronounced in conditions with a very small sample size (*N* = 30) and a small true correlation. For example, in the condition with *N* = 30, ρ = 0.30, and uniform prior distributions, the RMSE of the estimates produced by the EAP were only 71% as large as the estimates produced by the PML. This is an important finding because it clearly shows that, even with (diffuse) uniform distributions on the loadings and the correlation, using the EAP (obtained from MCMC) stabilizes the parameter estimates compared to the PML (or constrained ML) method.

To further understand the RMSE differences, we calculated the empirical standard deviation (*SD*) of the estimators across the 1000 replications within each cell. The results show that the estimates of the PML were consistently more variable (across the different prior specifications) than those of the EAP. For both estimators, PML and EAP, selecting a more informative prior distribution for the correlation (e.g., ν_ρ_ = 3 instead of ν_ρ_ = 1) had a large positive effect on the accuracy of the parameter estimates. By contrast, choosing a more informative prior distribution for the standardized loadings did not consistently influence the accuracy of the estimates of the latent correlation. Thus, adding information to the prior distribution for the parameter of interest was the only specification that helped to stabilize estimates of the latent correlation in small sample sizes.

**FIGURE 4:**
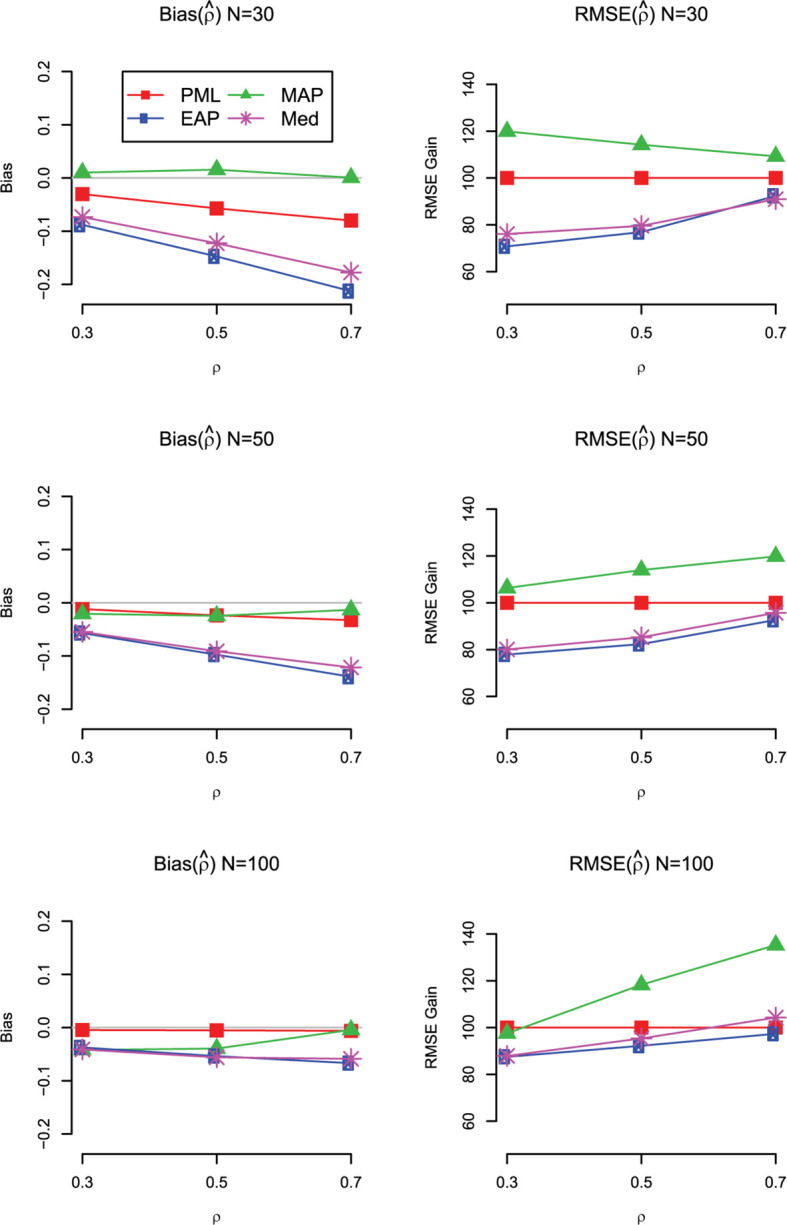
Simulation Study 1: Bias **(left panels)** and RMSE gain **(right panels)** of the estimators of the correlation (ρ) as a function of the sample size and the magnitude of the true correlation. For the RMSE gain, PML is used as a reference method; values smaller than 100 indicate that the RMSE of the respective method is lower than the RMSE of the reference method; PML = mode of joint posterior; MAP = mode of marginal posterior; Med = median of marginal posterior; EAP = mean of marginal posterior. Results are shown for models with uniform prior distributions for the correlation and the standardized loadings.

The main findings for bias and RMSE are summarized in Figure 4 for the case with uniform prior distributions on the standardized loadings and the correlation. We also show the results for the mode (MAP) and the median (Med) of the marginal posterior of ρ. As can be seen, the Med performed similar to the EAP but showed slightly larger RMSE values. By contrast, the MAP provided less accurate estimates of the correlation in terms of RMSE and was even outperformed by the PML in almost all conditions (except for *N* = 100 and ρ = 0.3).

Furthermore, we assessed the coverage rates for the PML and MCMC methods. As can be seen in Table 6, the PML method provided acceptable coverage rates with percentages close to the nominal 95% in conditions with *N* = 100. In addition, the coverage rates produced by the MCMC method were sometimes too low, even in conditions with *N* = 100. However, these low coverage rates can be explained by the fact that the MCMC method was also more biased in these conditions.

**TABLE 6:** Simulation study 1: coverage rates of the latent correlation for penalized maximum likelihood and Markov chain monte carlo methods with different correctly specified prior distributions as a function of the magnitude of the true correlation (ρ) and sample size.

			PML			MCMC		
			ρ			ρ		
*N*	ν_ρ_	ν_λ_	0.3	0.5	0.7	0.3	0.5	0.7
30	0	0	**85.8**	**87.0**	91.1	**98.9**	**98.6**	98.0
	0	1	**87.6**	91.2	96.8	97.5	97.9	97.9
	0	3	**88.5**	94.1	97.5	97.3	97.7	**98.1**
	1	0	**88.9**	**90.3**	94.7	**99.2**	98.0	**89.3**
	1	1	**90.0**	93.6	97.7	**98.2**	95.9	**84.3**
	1	3	**90.7**	94.2	**98.3**	**98.2**	95.5	**83.3**
	3	0	92.8	95.6	96.8	**99.6**	**99.5**	**99.4**
	3	1	95.2	97.0	**98.3**	**99.6**	**99.0**	**98.5**
	3	3	97.5	97.8	**98.8**	**99.2**	**99.1**	98.0
50	0	0	**86.6**	**90.1**	93.1	97.1	**98.2**	97.4
	0	1	**88.4**	92.7	95.7	95.5	96.1	96.7
	0	3	**90.1**	93.6	97.4	94.9	95.7	97.0
	1	0	**87.9**	**90.0**	96.5	97.5	96.9	**89.4**
	1	1	**89.9**	92.4	**98.2**	96.2	95.6	**87.3**
	1	3	92.0	91.8	**98.8**	95.6	93.8	**85.3**
	3	0	91.8	96.4	97.7	**98.8**	**98.8**	**98.3**
	3	1	95.1	97.2	**98.7**	98.0	**98.3**	96.5
	3	3	96.7	97.0	**99.3**	97.9	97.5	95.5
100	0	0	**89.9**	92.7	97.0	95.6	95.7	96.8
	0	1	92.1	94.8	97.0	95.3	96.1	97.2
	0	3	92.8	95.4	97.5	94.8	96.4	96.9
	1	0	91.0	92.3	94.4	96.8	96.7	**89.9**
	1	1	92.3	92.5	94.6	96.1	94.9	**88.5**
	1	3	93.0	91.0	95.1	95.5	94.5	**87.8**
	3	0	93.8	95.8	95.1	97.3	**98.5**	97.0
	3	1	95.5	96.3	95.1	97.1	97.8	96.4
	3	3	95.8	96.1	95.8	96.6	98.0	95.1

*PML = mode of joint posterior (obtained from penalized maximum likelihood) with standard errors (calculated from observed information matrix); MCMC = Bayesian Credible Interval (BCI) based on MCMC; ρ = true latent correlation; ν_ρ_ = prior sample size for latent correlation; ν_λ_ = prior sample size for standardized loading. Coverage rates smaller than 91% or larger than 98% are printed in bold.*

**FIGURE 5:**
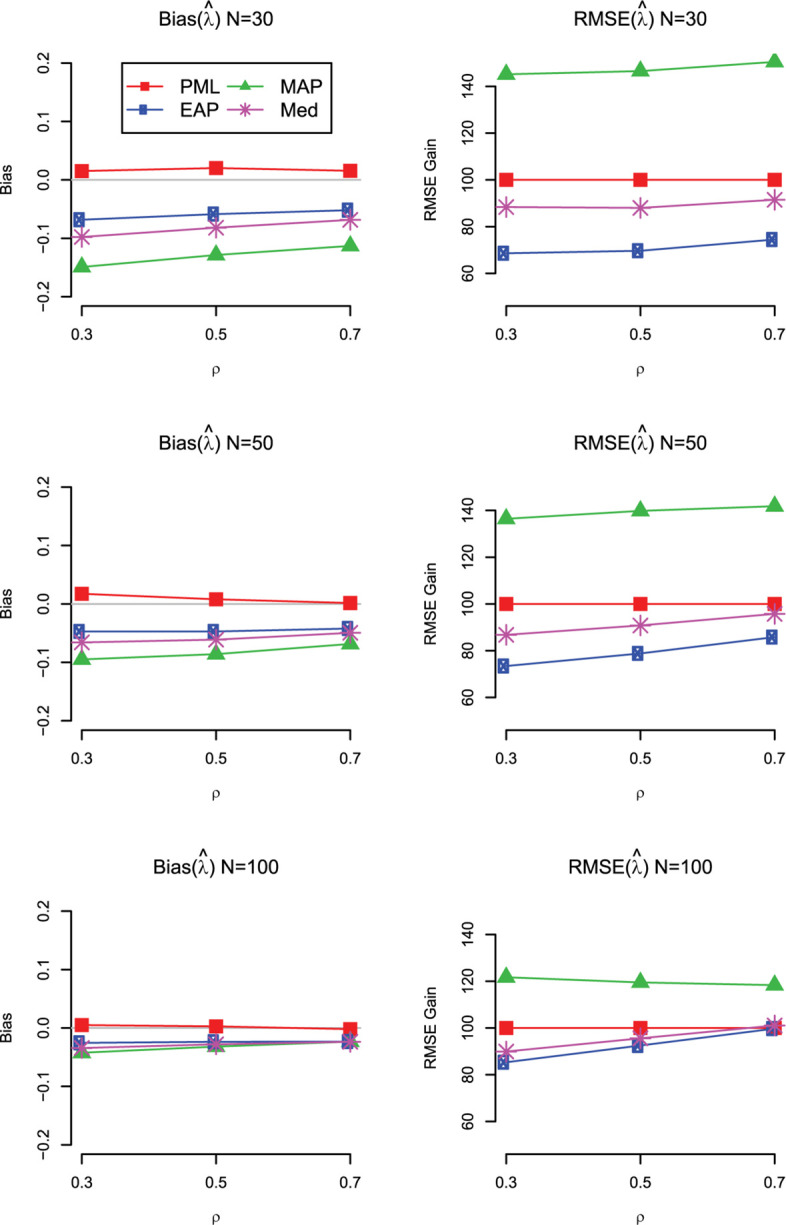
Simulation Study 1: Bias **(left panels)** and RMSE gain **(right panels)** of the estimators of a loading (λ) as a function of the sample size and the magnitude of the true correlation. For the RMSE gain, PML is used as a reference method; values smaller than 100 indicate that the RMSE of the respective method is lower than the RMSE of the reference method; PML = mode of joint posterior; MAP = mode of marginal posterior; Med = median of marginal posterior; EAP = mean of marginal posterior. Results are shown for models with uniform prior distributions for the correlation and the standardized loadings.

Finally, we also investigated the bias and RMSE of the different Bayesian estimates for a standardized loading. The main results are summarized in Figure 5 for the case with uniform prior distributions (for the detailed results, see Supplementary 3 at https://doi.org/fwr7). Overall, the findings are in line with the results for the correlation. The EAP produced the most accurate estimates of the loadings in terms of RMSE across the investigated conditions, even though the estimates were slightly negatively biased, particularly in conditions with *N* = 30. Interestingly, with smaller sample sizes, the univariate mode (MAP) was clearly outperformed by the multivariate mode (PML). Further simulation research should compare the different Bayesian point estimates for more extreme values of the loading (i.e., standardized loading of 0.3 or 0.9). It is possible that with smaller or larger loading values, the bias introduced by the EAP outweighs the gains in variability, resulting in different conclusions about the overall accuracy of the different Bayesian point estimates.

#### Bayesian Estimation With Misspecified Priors

We also assessed the impact of misspecified prior distributions. Table 7 shows the bias and RMSE for *N* = 30 and *N* = 100. The main findings can be summarized as follows. First, even in the case of misspecified prior distributions, the EAP outperformed the PML in terms of the RMSE and provided more accurate parameter estimates across most conditions and prior specifications. Second, a misspecified prior distribution for the loading had only a small and sometimes even positive effect on the RMSE. One possible explanation is that we only included misspecified priors that overestimated the true size of the loading (i.e., μ_λ_ = 0.80). Overestimating the reliability of the indicators by assuming a large positive loading comes close to a manifest approach that ignores the unreliability of scale scores when calculating the correlation. However, with small sample sizes, it has been shown that a manifest approach can produce more accurate estimates of structural relationships than a latent approach that corrects for measurement error (e.g., Lüdtke et al., 2008; Ledgerwood and Shrout, 2011; Savalei, 2019). Third, for the prior distribution of the correlation, the results clearly show that overestimating the true size of the latent correlation (i.e., μ_ρ_ = 0.80) had a more negative impact on the accuracy of the estimates in terms of RMSE than underestimating the size of the true correlation (i.e., μ_ρ_ = 0.20). More importantly, choosing a small correlation of 0.20 as a prior guess for the prior distribution, even though misspecified, produced more accurate estimates of the correlation than the Bayesian approach with uniform priors on the loadings and the correlation, particularly when the sample size was *N* = 30. Thus, a conservative approach that uses smaller prior guesses for the latent correlation seems to be a promising strategy when the goal is to stabilize the estimates of the latent correlations with weakly informative prior distributions (i.e., prior sample sizes of 1 or 3).

**TABLE 7:** Simulation study 1: bias and RMSE for the latent correlation as a function of different misspecified prior distributions and the sample size.

				Bias					RMSE			
			μ_λ_ = 0.5			μ_λ_ = 0.8		μ_λ_ = 0.5			μ_λ_ = 0.8	
Meth	μ_ρ_	ν_ρ_	ν_λ_ = 0	ν_λ_ = 1	ν_λ_ = 3	ν_λ_ = 1	ν_λ_ = 3	ν_λ_ = 0	ν_λ_ = 1	ν_λ_ = 3	ν_λ_ = 1	ν_λ_ = 3
			*N = 30*									
PML	0	0	–0.038	0.009	0.019	−**0.139**	–0.022	0.391	**0**.**393**	0.379	0.361	0.367
	0.5	1	**0.161**	**0.196**	**0.201**	–0.008	**0.119**	0.388	**0**.**396**	**0**.**397**	0.325	0.344
	0.5	3	**0.094**	**0.120**	**0.123**	–0.019	**0.089**	0.281	0.281	0.278	0.261	0.263
	0.2	1	–0.027	0.021	0.026	−**0.114**	–0.002	0.320	0.322	0.317	0.323	0.318
	0.2	3	−**0.086**	–0.040	–0.039	−**0.136**	–0.046	0.269	0.259	0.255	0.290	0.272
	0.8	1	**0.297**	**0.328**	**0.347**	**0.112**	**0.237**	**0**.**432**	**0**.**444**	**0**.**447**	0.358	0.384
	0.8	3	**0.337**	**0.360**	**0.392**	**0.153**	**0.271**	**0**.**427**	**0**.**436**	**0**.**453**	0.329	0.373
EAP	0	0	−**0.141**	−**0.070**	–0.050	−**0.180**	−**0.103**	0.303	0.307	0.314	0.313	0.314
	0.5	1	**0.131**	**0.151**	**0.161**	–0.035	**0.056**	0.222	0.264	0.276	0.247	0.268
	0.5	3	0.004	0.038	0.048	−**0.066**	0.011	0.157	0.188	0.196	0.199	0.202
	0.2	1	−**0.121**	−**0.071**	−**0.058**	−**0.163**	−**0.088**	0.242	0.248	0.252	0.277	0.268
	0.2	3	−**0.178**	−**0.140**	−**0.131**	−**0.192**	−**0.131**	0.245	0.231	0.228	0.266	0.243
	0.8	1	**0.360**	**0.340**	**0.330**	**0.145**	**0.222**	0.387	0.377	0.375	0.282	0.319
	0.8	3	**0.309**	**0.320**	**0.327**	**0.151**	**0.231**	0.334	0.346	0.352	0.252	0.300
			*N = 100*									
PML	0	0	–0.011	0.002	0.006	–0.039	–0.024	0.203	0.196	0.194	0.194	0.181
	0.5	1	**0.089**	**0.090**	**0.090**	0.031	0.035	**0**.**273**	**0**.**261**	**0**.**255**	**0**.**231**	**0**.**212**
	0.5	3	0.032	0.042	0.046	0.002	0.014	0.183	0.180	0.179	0.173	0.162
	0.2	1	–0.008	0.004	0.008	–0.034	–0.020	0.190	0.184	0.183	0.186	0.173
	0.2	3	–0.036	–0.024	–0.020	–0.055	–0.040	0.172	0.165	0.164	0.176	0.164
	0.8	1	**0.188**	**0.185**	**0.163**	**0.091**	**0.082**	**0**.**332**	**0**.**324**	**0**.**302**	**0**.**262**	**0**.**234**
	0.8	3	**0.218**	**0.216**	**0.203**	**0.135**	**0.118**	**0**.**340**	**0**.**332**	**0**.**321**	**0**.**286**	**0**.**253**
EAP	0	0	−**0.059**	–0.034	–0.024	−**0.113**	−**0.069**	0.191	0.187	0.187	**0**.**207**	0.185
	0.5	1	0.039	0.054	**0.059**	–0.053	–0.016	0.199	**0**.**207**	**0**.**208**	0.195	0.180
	0.5	3	–0.016	0.003	0.011	−**0.069**	–0.030	0.155	0.156	0.157	0.171	0.154
	0.2	1	−**0.062**	–0.039	–0.029	−**0.109**	−**0.066**	0.176	0.171	0.171	0.197	0.174
	0.2	3	−**0.095**	−**0.072**	−**0.061**	−**0.128**	−**0.086**	0.174	0.163	0.160	0.195	0.170
	0.8	1	**0.191**	**0.182**	**0.178**	0.038	**0.059**	**0**.**278**	**0**.**270**	**0**.**266**	**0**.**213**	**0**.**204**
	0.8	3	**0.177**	**0.173**	**0.176**	0.041	**0.064**	**0**.**248**	**0**.**249**	**0**.**253**	0.195	0.189

*RMSE = root mean square error. PML = penalized maximum likelihood (mode of joint posterior); EAP = mean of marginal posterior; μ_ρ_ = prior guess for latent correlation; ν_ρ_ = prior sample size for latent correlation; μ_λ_ = prior guess for loading ν_λ_ = prior sample size for loading; biases smaller than –0.05 or larger than 0.05 are printed in bold. For each sample size condition, RMSE values larger than the RMSE for the PML method with uniform priors on loadings (μ_λ_ = 0.5, ν_λ_ = 0) and the correlation (μ_ρ_ = 0, ν_ρ_ = 0) are printed in bold. The true correlation and loadings were set to ρ = 0.50 and λ = 0.50, respectively.*

## Simulation Study 2

The previous simulation study assumed that the observed variables were multivariate normally distributed. However, the true distribution is rarely known for real data, and the CFA will likely be misspecified to some extent. In Simulation Study 2, we investigate how robust the Bayesian approach is against the misspecification of the distributional assumptions. More specifically, we consider the case of observed variables that are linearly related but have non-normal marginal distributions (Foldnes and Olsson, 2016). Again, we compared the different Bayesian point estimates obtained from the joint posterior (PML) or the marginal posterior distribution (MAP, EAP, and Med). As a benchmark, we also included ML approaches that are based on robust estimation approaches (Yuan et al., 2004; Yuan and Zhang, 2012). For further comparisons, we also considered an unweighted least squares (ULS) estimation method (Browne, 1974). Limited information methods such as ULS are expected to be more robust in modeling violations than ML estimators (MacCallum et al., 2007).

### Simulation Model and Conditions

The data-generating model was again a two-factor CFA model with six observed variables. We generated a covariance structure (see Equation 2) that followed a CFA model with parallel and standardized loadings of 0.50 and a variance of one for the observed variables. The procedure of Foldnes and Olsson (2016) was used to generate six observed variables that preserved the covariance structure and had a prespecified level of skewness and kurtosis for the marginal distributions of observed variables. Six different combinations of skewness and kurtosis values were chosen to implement a range of non-normal distributions for the observed variables: 0/0 (skewness/excess kurtosis), 0/3, 0/7, 1/3, 1/7, and 2/7. Again, we manipulated the latent correlation between the two factors (ρ = 0.10, 0.30, 0.50, 0.70, and 0.90) and the sample size (*N* = 30, 50, 100, and 500). For each of the 5 × 5 × 4 = 100 conditions, we generated 1,000 simulated data sets.

### Analysis Models

Each of the simulated data sets was analyzed with a two-factor CFA in which the loadings were freely estimated, and the variances of the two factors were set to one. We used PML estimation with a uniform prior distribution on the standardized loadings and the correlation. In addition, we included a robust version of PML (PMLR) in which the sufficient statistics $\bar{\text{x}}$ and **S** were replaced by a robust sample mean vector ${\bar{\text{x}}}_{\text{rob}}$ and a robust sample covariance matrix **S**_rob_ that were obtained with the R package rsem (Yuan and Zhang, 2012). The robust estimation procedure provides Huber-Type M-estimates of means and covariances and has been shown to produce more efficient parameters, particularly for distributions with heavy tails (Yuan et al., 2004). For comparison purposes, we also included an ULS estimation method. The ULS estimate is defined as:

(34) $${\hat{\theta }}_{\text{ULS}}=\underset{\theta }{\arg\,\min}\text{tr}\,\left\{{\left(\text{S}-\Sigma \,\left(\theta \right)\right)}^{\text{T}}\,\left(\text{S}-\Sigma \,\left(\theta \right)\right)\right\}$$

The ULS method was also specified with robustly estimated means and covariances (ULSR; Yuan et al., 2004). Finally, the MCMC method was applied to obtain the mode (MAP), mean (EAP), and median (Med) of the marginal posterior distributions. We specified uniform distributions for the standardized loadings and the correlation. For the standard deviations of the indicator variables, we used improper prior distributions that are constant for all conditions of the simulation and all (Bayesian) analysis models. The R code for the data-generating model and the different analysis models is provided in Supplementary 4 at https://doi.org/fwr7.

### Results

Table 8 shows the bias and RMSE for the different estimators of the correlation for conditions with a true correlation of ρ = 0.50 (see Supplementary 5 for detailed information about the other conditions). We again report RMSE gain with PML as the reference method (i.e., values larger/smaller than 100 indicate that the RMSE for the respective method is larger/smaller than for PML). Overall, the results confirm the previous findings that the EAP and Med produce (negatively) bias estimates of the correlation. However, with smaller sample sizes (*N* ≤ 50), the estimates of the EAP and the Med were also more accurate in terms of the RMSE gain. When the variables strongly deviated from normality and the sample size was large, the robust estimation approaches (PMLR, ULSR, and ULSR) were slightly more efficient (i.e., smaller SD of the parameter estimates) than the different Bayesian point estimates. However, the results also reveal that, for moderate deviations from normality, the conclusions about the performance of the different Bayesian point estimates are relatively robust against distributional misspecifications. In addition, it should be mentioned that the multivariate mode (PML) consistently outperformed the univariate mode (MAP) across all conditions.

**TABLE 8:** Simulation study 2: bias and RMSE for the latent correlation as a function of the distribution of the observed variables (skewness and kurtosis) and the sample size.

		Bias							RMSE Gain						
skew/kurt	*N*	PML	PMLR	ULS	ULSR	MAP	Med	EAP	PML	PMLR	ULS	ULSR	MAP	Med	EAP
0/0	30	−**0.065**	−**0.067**	−**0.010**	−**0.011**	−**0.003**	−**0.131**	−**0.154**	100	**101**	99	**101**	**116**	80	77
	50	–0.019	–0.020	0.016	0.017	–0.018	−**0.085**	−**0.092**	100	**101**	96	98	**114**	85	82
	100	–0.014	–0.016	0.008	0.008	−**0.051**	−**0.063**	−**0.061**	100	**101**	94	95	**116**	95	92
	500	–0.002	–0.002	0.002	0.001	–0.011	–0.012	–0.013	100	**101**	99	100	**102**	**101**	**101**
0/3	30	–0.038	–0.033	0.000	0.007	0.027	−**0.106**	−**0.130**	100	100	**101**	98	**119**	80	77
	50	–0.042	–0.036	0.002	0.001	–0.037	−**0.102**	−**0.109**	100	98	97	94	**108**	84	81
	100	–0.007	–0.004	0.015	0.016	–0.041	−**0.058**	−**0.056**	100	94	96	91	**117**	95	92
	500	–0.003	–0.005	0.001	–0.002	–0.011	–0.013	–0.014	100	97	99	95	**104**	**101**	**101**
0/7	30	–0.043	–0.024	0.007	0.016	0.024	−**0.108**	−**0.133**	100	95	**101**	98	**116**	82	79
	50	–0.021	–0.011	0.019	0.021	–0.021	−**0.085**	−**0.092**	100	96	97	93	**115**	87	84
	100	–0.004	–0.004	0.018	0.013	–0.037	−**0.054**	−**0.052**	100	92	98	89	**117**	94	91
	500	–0.003	–0.002	0.001	0.002	–0.012	–0.013	–0.014	100	92	99	91	**101**	100	**101**
1/3	30	–0.048	–0.046	0.003	0.004	0.026	−**0.110**	−**0.136**	100	99	**103**	99	**119**	81	79
	50	–0.023	–0.018	0.016	0.019	–0.019	−**0.086**	−**0.093**	100	98	95	94	**111**	82	79
	100	–0.006	–0.012	0.013	0.008	–0.042	−**0.055**	−**0.054**	100	99	94	93	**116**	96	92
	500	0.002	–0.002	0.005	0.001	–0.006	–0.008	–0.009	100	97	99	96	**103**	**101**	**101**
2/7	30	–0.015	–0.009	0.033	0.029	**0.064**	−**0.078**	−**0.105**	100	95	99	93	**114**	77	74
	50	–0.017	–0.017	0.017	0.001	–0.014	−**0.079**	−**0.086**	100	94	97	94	**113**	87	84
	100	–0.003	–0.015	0.015	0.001	–0.035	−**0.051**	–0.049	100	93	98	89	**117**	96	93
	500	0.003	–0.014	0.007	–0.011	–0.006	–0.007	–0.008	100	95	99	94	**103**	100	100

*RMSE = root mean square error. PML = penalized maximum likelihood; PMLR = penalized maximum likelihood with robustly estimated covariance matrix; ULS = unweighted least squares; ULSR = unweighted least squares with robustly estimated covariance; MAP = mode of marginal posterior; Med = median of marginal posterior; EAP = mean of marginal posterior; μ_ρ_ = prior guess for latent correlation; ν_ρ_ = prior sample size for latent correlation; skew = skewness; kurt = kurtosis; Biases smaller than –0.05 or larger than 0.05 are printed in bold. For the RMSE gain, the RMSE of PML estimation is used as a reference method; values smaller than 100 indicate that the RMSE of the respective method is lower than the RMSE of the reference method. The true correlation and loadings were set to ρ = 0.50 and λ = 0.50, respectively.*

Furthermore, we obtained similar results for the estimates of the loadings (see Supplementary 6); that is, the estimates produced by the EAP and Med were slightly biased but overall more accurate in terms of RMSE than the other approaches. Again, the performance differences between the Bayesian point estimates were relatively robust against deviations from normality, and larger sample sizes were needed to show the gains in efficiency for the robust estimation approaches (with the exception that the ULS method performed less favorably with *N* = 500).

## Discussion

In this article, we showed that a Bayesian approach can stabilize the parameter estimates of a CFA model in small sample size conditions. We discussed different Bayesian point estimators—the mode (PML) of the joint posterior distribution and the mean (EAP), median (Med), or mode (MAP) of the marginal posterior distribution—and evaluated their performance in two simulation studies from a frequentist point of view. The results showed that the EAP outperformed the PML in terms of RMSE and produced more accurate estimates of latent correlations in many conditions. These performance gains can be explained by the fact that the EAP pulls large estimates toward zero (i.e., shrinkage effect), resulting in less variable estimates of the correlation. However, there is a turning point at which, with a larger true correlation, the EAP is less accurate than the PML because the bias introduced by the shrinkage effect outweighs the gains in efficiency (see Choi et al., 2011). As expected, with larger sample sizes, the differences between the Bayesian point estimates vanished, and the different Bayesian estimators performed similarly. We also suggested the four-parameter beta distribution as a prior distribution for loadings and correlations and argued that it could often be advantageous to choose a parameterization in which the main parameters of interest are bounded (Muthén and Asparouhov, 2012; Merkle and Rosseel, 2018). Another finding of our simulation study was that selecting weakly informative four-parameter beta distributions as priors helped stabilize parameter estimates (e.g., Depaoli and Clifton, 2015; van Erp et al., 2018). Importantly, this was also the case when the prior was mildly misspecified.

The main limitation of our simulation study is that we used a very simple CFA model with only two latent factors and a small number of items with no cross-loadings (i.e., simple structure). It would be straightforward to extend the discussed approaches to models with more latent factors. In constrained ML estimation and PML estimation, appropriate determinant constraints could be implemented to ensure the positive definiteness of the correlation matrix of latent variables (Wothke, 1993; Rousseeuw and Molenberghs, 1994). For the MCMC method, determinant constraints could be introduced in the Metropolis-Hastings step to check for the positive definiteness of the correlation matrix (see Browne, 2006).

In addition, we only assessed the quality of statistical inferences (i.e., coverage rates) with normally distributed variables. It would be an interesting topic for future research also to investigate robust estimation approaches for Bayesian CFA models. First, one could use robustly estimated covariance matrices as input for Bayesian CFA models. In this case, robust standard errors must also be applied in Bayesian estimation because the model is misspecified (Müller, 2013; Walker, 2013; Bissiri et al., 2016). Second, models with more flexible distributions for the latent factors and residuals could be applied (Lin et al., 2018). For example, Zhang et al. (2014) proposed a Bayesian factor analysis model with scaled *t*-distributions (and freely estimated degrees of freedom) that are less sensitive to outlier values.

The results of the present study could be extended into several directions. First, it would be interesting to explore further the potential of PML estimation for CFA models in challenging data constellations (e.g., small samples, complex models; Rosseel, 2020). PML estimation seems to be particularly promising when researchers do not need full access to the posterior distribution and are only interested in obtaining stable point estimates for the parameters of interest. In contrast to simulation-based MCMC techniques, which can be slow and challenging to implement, PML estimation shares the advantage of traditional ML estimation that a deterministic optimization of the log-posterior is performed with clear convergence criteria and reasonable computational efficiency (Cousineau and Helie, 2013). In Simulation Study 1, for example, the average run time was about 2 min for MCMC but only two seconds for PML. The run time differences could be considerably larger with more complex models (Chung et al., 2013). Second, data sets in psychological research often have a multilevel structure (e.g., individuals are nested within clusters/groups) and, in many applications, it is of interest to analyze relationships among latent constructs at both levels of analysis (e.g., individual level and group level; Heck and Thomas, 2015). However, a notable finding in the multilevel literature is that a substantial number of groups is needed to obtain stable parameter estimates of group-level relationships (Lüdtke et al., 2011; Li and Beretvas, 2013; Kelava and Brandt, 2014; Can et al., 2015). Thus, an important topic for future research could be to extend the Bayesian approaches discussed here to multilevel CFA models (Kim et al., 2016). Finally, it would be interesting to compare the different Bayesian estimators to other approaches that have been suggested as solutions for estimation problems in small sample size conditions (Rosseel, 2020). For example, a two-step approach, such as factor score regression, has been suggested as a robust alternative to SEMs in challenging data constellations (Smid and Rosseel, 2020). Besides, alternative error correction approaches could be used that introduce lower bounds to circumvent small estimates of reliability in order to stabilize the estimation of latent correlations (Grilli and Rampichini, 2011). Using these lower bounds, *l* can be translated into a uniform distribution of standardized loadings on the interval [*l*, 1]. However, using lower bounds for the indicator-specific reliability larger than zero possibly introduces too much information. Besides, parameter estimates could be pretty sensitive to the subjective choice of lower bounds.

To conclude, this article showed that the Bayesian approach has great potential for estimating CFA models with small sample sizes. Using simulated data, we showed that the four-parameter beta distribution can be used as a prior distribution for standardized loadings and latent correlations to stabilize parameter estimates in challenging data constellations. However, in real applications, the specification of prior distributions should be accompanied by a sensitivity analysis that tests how sensitive the resulting parameter estimates are to different specifications of prior information (Depaoli and van de Schoot, 2017).

## Data Availability Statement

The original contributions presented in the study are included in the article/Supplementary Material, further inquiries can be directed to the corresponding author/s.

## Author Contributions

All authors listed have made a substantial, direct and intellectual contribution to the work, and approved it for publication.

## Conflict of Interest

The authors declare that the research was conducted in the absence of any commercial or financial relationships that could be construed as a potential conflict of interest.

## Footnotes

1 https://shortly.cc/ArTE6

2 In the joint estimation approach, the joint likelihood $L\,\left(\theta ,\eta |\text{X}\right)=\prod_{i=1}^{n}f\,\left({\text{x}}_{i};\nu +\Lambda \,\eta_{i},\Omega \right)\cdot f\,\left(\eta_{i};0,\Phi \right)$ is considered, and the MCMC method generates samples for θ and η (*t* = 1,…,*T*): θ^(*t*)^,η^(*t*)^*p*(θ,η|**X**)∝*L*(θ,η|**X**)⋅π(θ). Note that the joint estimation approach needs the raw data **X** instead of the sufficient statistic **S** (see Choi and Levy, 2017).

3 This was confirmed by preliminary simulation studies in which we also included unconstrained ML estimation with the reference variable method (i.e., first loading fixed to 1). The parameterization with bounded parameters clearly outperformed the parameterization with freely estimated variances and covariance of the latent variables in terms of estimation problems (e.g., convergence) and quality of parameter estimates (e.g., RMSE).

4 For constrained ML estimation, it can be shown that this parameterization of the CFA model with standard deviations that are constrained to be positive and loadings that are restricted to the interval [0, 1] is (analytically) equivalent to a parameterization in which the loadings and error variances of the unstandardized solution are constrained to be positive and the variances of the latent factors are set to one. Thus, using the parameterization of the CFA model in Equation 21 for constrained ML estimation should – in theory – provide the same results as a CFA model with the corresponding inequality constraints on the loadings and error variances.

5 Alternatively, if a researcher is not willing to make assumptions about the sign of the loading (i.e., loadings are assumed to be positive), the loadings can be restricted to [−1, 1]. However, if this specification is chosen, researchers need to define one marker item for which loadings must be restricted to [0, 1]. Otherwise, sign switching issues can occur in the MCMC algorithm. In preliminary simulations, we investigated the effect of both restrictions. If the loadings of the data-generating model were all positive, the performance for the two variants of restrictions was very similar.

6 For this illustration, we further reduced the number of estimated parameters, assumed that the indicators had a variance of one, and also fixed the variances of the indicators in the analysis model to one. The main purpose of the illustration was to demonstrate the differences between the mode from the joint posterior and the EAP that is obtained from the marginal posterior distribution. A more systematic and realistic evaluation of the different estimators is provided in the two main simulations.

7 For example, in the context of state-trait models, Lüdtke et al. (2018) found in simulation studies that PML (obtained from constrained ML estimation) was clearly outperformed by the MAP (obtained from MCMC) in terms of the accuracy of the estimated variance components (e.g., stable trait variance, state variance). This can be explained by the fact that using marginal distributions stabilizes point estimates if model parameters are substantially correlated.

8 For constrained ML estimation, we also included the equivalent parameterization (see Equation 20) in which the loadings and residual variances were constrained to be positive and the latent correlation was restricted to the interval [−1, 1]. As expected, the results for both parameterizations were virtually identical in every replication (results were numerically identical in 95.5% of the replications for *N* = 30, in 98.4% for *N* = 50, and in 99.9% for *N* = 100). Moreover, the parameterization using standardized instead of unstandardized loadings performed slightly better in terms of RMSE.
